# MOLA: a bootable, self-configuring system for virtual screening using AutoDock4/Vina on computer clusters

**DOI:** 10.1186/1758-2946-2-10

**Published:** 2010-10-28

**Authors:** Rui MV Abreu, Hugo JC Froufe, Maria João RP Queiroz, Isabel CFR Ferreira

**Affiliations:** 1 CIMO-ESA, Instituto Politécnico de Bragança, Campus de Sta Apolónia, Apartado 1172, 5301-855 Bragança, Portugal; 2 Instituto de Biotecnologia e Bioengenharia, Centro de Genética e Biotecnologia, Universidade de Trás-os-Montes e Alto Douro (CGB-UTAD/IBB), 5001-801, Vila Real, Portugal; 3 Centro de Química, Universidade do Minho, Campus de Gualtar 4710-057 Braga, Portugal

## Abstract

**Background:**

Virtual screening of small molecules using molecular docking has become an important tool in drug discovery. However, large scale virtual screening is time demanding and usually requires dedicated computer clusters. There are a number of software tools that perform virtual screening using AutoDock4 but they require access to dedicated Linux computer clusters. Also no software is available for performing virtual screening with Vina using computer clusters. In this paper we present MOLA, an easy-to-use graphical user interface tool that automates parallel virtual screening using AutoDock4 and/or Vina in bootable non-dedicated computer clusters.

**Implementation:**

MOLA automates several tasks including: ligand preparation, parallel AutoDock4/Vina jobs distribution and result analysis. When the virtual screening project finishes, an open-office spreadsheet file opens with the ligands ranked by binding energy and distance to the active site. All results files can automatically be recorded on an USB-flash drive or on the hard-disk drive using VirtualBox. MOLA works inside a customized Live CD GNU/Linux operating system, developed by us, that bypass the original operating system installed on the computers used in the cluster. This operating system boots from a CD on the master node and then clusters other computers as slave nodes via ethernet connections.

**Conclusion:**

MOLA is an ideal virtual screening tool for non-experienced users, with a limited number of multi-platform heterogeneous computers available and no access to dedicated Linux computer clusters. When a virtual screening project finishes, the computers can just be restarted to their original operating system. The originality of MOLA lies on the fact that, any platform-independent computer available can he added to the cluster, without ever using the computer hard-disk drive and without interfering with the installed operating system. With a cluster of 10 processors, and a potential maximum speed-up of 10x, the parallel algorithm of MOLA performed with a speed-up of 8,64× using AutoDock4 and 8,60× using Vina.

## Background

Intermolecular interactions between proteins and small ligands play essential roles in several life processes including enzyme catalysis, gene expression and regulation of metabolic pathways. Understanding these interactions is thus critical for pharmaceutical and functional food industries particularly in the process of drug discovery [1]. Molecular docking is an *in silico *tool that predicts how a ligand (substrate or drug candidate) interacts with a receptor usually by predicting the binding free energy and the three-dimensional structure of the ligand-receptor complex. The use of molecular docking to search large compound databases for possible ligands of a protein receptor is usually termed virtual screening and has been successfully applied in several therapeutic programs at the lead discovery stage [2]. Several molecular docking tools, using different approaches, have been developed [1]. The software presented in this article uses AutoDock4 (version 4.2) [3] and Vina (AutoDock Vina) [4] as docking engine.

AutoDock4 is acknowledged to be one of the most reliable and broadly used molecular docking tool [5], with several examples of accurate docking predictions already published [6,7]. AutoDock4 uses genetic algorithms and the results are given as estimates of free-energy change (ΔG) upon ligand binding and as a prediction of the ligand-receptor complex three-dimensional conformation. A major difficulty for virtual screening using AutoDock4 is the time necessary for each molecular docking experiment. Considering that compound databases usually can have up to hundreds of thousands of small molecule it is easy to see that, to perform virtual screening, High Performance Computing (HPC) systems and tools are required.

Vina is a new software for molecular docking and was designed to be compatible with AutoDock4 file formats. Vina uses a different docking algorithm with a gradient optimization method in its local optimization procedure [4]. A comparison with AutoDock4 showed that Vina achieved approximately a two orders of magnitude speed-up with significant improvement of the accuracy of the binding mode prediction [4]. Another advantage of Vina is that some preparation steps need for AutoDock4 like atom grid map calculation and result clustering are done automatically. Although promising Vina is still a recent tool and needs further testing for users around the world.

Before using AutoDock4, several steps are required including ligand preparation, receptor preparation and atom grid maps calculations. A number of software tools have been developed to facilitate the use of AutoDock4 by providing graphical user interfaces (GUI), including AutoDockTools (ADT) [8], BDT [9] and Pymol AutoDock/Vina plug-in [10]. These tools greatly reduce AutoDock4 and Vina learning curve but, although providing some automated features for molecular docking and virtual screening, were not developed to be used with computer clusters. DOVIS [11,12] is another tool for virtual screening with AutoDock4 that includes a GUI and automates most of the pre-docking steps required for docking with AutoDock4. Still DOVIS was designed to be used with large dedicated Linux computer clusters usually with integrated queuing systems such as LSF (Load Sharing Facility) or PBS (Portable Batch System). All these virtual screening tools using AutoDock4 have two requirements: access to dedicated HPC Linux clusters and strong computer skills in Linux systems. Many research groups with interest in virtual screening fail these requirements. Our research group lacked access to a dedicated HPC Linux cluster but had access to a number of multi-platform (with Windows, Linux and Macintosh operating systems) heterogeneous computers (different processing capabilities) that we could use, provided we did not interfere with the data on the hard-disks and the installed operating system. As we believe this situation is rather common for other research groups we then set out to develop MOLA, a tool that uses the processing power of these computers for virtual screening without compromising their every day use. Also, as far as we know, there is no tool available for virtual screening in computer clusters using Vina.

In this paper we present MOLA, a tool with a simple GUI that automates: ligand preparation, distribution of AutoDock4/Vina jobs and result analysis. MOLA works inside a customized Live-CD (Compact Disk) GNU/Linux operating system that handles the computer cluster and was designed to work with an heterogeneous set of computers. This customized operating system boots from a CD without ever using the computer hard-disk drive and the installed operating system. When a virtual screening project finishes the computers can be restored to the installed operating system by simply removing the CD and restarting. All result files can be conveniently stored on a USB (Universal Serial Bus)-flash drive or on the computer hard-disk drive by using the VirtualBox software [13]. Special care was taken to provide a detailed step-by-step tutorial for MOLA (Additional file 1). MOLA is not intended for large clusters rather his main strengths are: (1) ease-of-use for users with little knowledge on Linux systems, (2) facility to integrate a heterogeneous set of computers and (3) the ability to use non-dedicated computers that can be easily restored to their original operating system.

### Implementation

MOLA is made available integrated on a customized Live-CD GNU/Linux operating system. This means that the user needs to download the complete operating system (available as a MOLA.iso file), burn it to a CD and then restart the master computer from the CD. Once the customized operating system is initiated all files and software packages need to use MOLA and to perform the MOLA tutorial are automatically available on the \home\user folder. These files include: MOLA scripts for AutoDock4 (MOLA-AD4) and Vina (MOLA-Vina), the NCI (National Cancer Institute) Diversity Set 2 compound database [14], Vina, AutoDock4, AutoGrid4, AutoDockTools and Pdb-tools. The customized operating system is installed in the RAM (Random Access Memory) memory instead of the hard-disk drive insuring that the installed operating system is not used.

The customized Live-CD GNU/Linux operating system was prepared by re-mastering the Pelican HPC GNU/Linux distribution [15] and can be considered a new GNU/Linux distribution for virtual screening with AutoDock4 and/or Vina. Pelican HPC was in turn developed using the Live Debian Linux distribution [16]. The customized operating system provides the frame work for setting up a cluster of machines for parallel processing using the LAM-MPI (LAM-MPI is a message passing interface specification used for parallel computing) and MPICH implementations of MPI [17].

### Cluster assembly

To assemble the cluster, the computer used as master node was first booted from the CD and the computer slave nodes were then booted via ethernet connections. Every service needed for LAM-MPI is configured automatically and all computers share a common directory, which is created on the master node using the NFS (network file system) protocol. This system assumes that the computers support PXE (Preboot Execution Environment) boot a standard feature for recent computers. If any of the slave nodes doesn't support PXE it is possible to use gPXE, an implementation of the PXE specification for network booting [18].

For this work we assembled clusters with 2, 6 and 10 Intel 2.8 GHz processors (CPUs) using 1, 3 and 5 Dual-Core computers respectively, with 1 Gigabyte of RAM memory. The performance of these clusters was compared to a system using only one CPU, referred below as serial test. Speed-up was calculated according to formula *Sp = T1/Tp; *were *p *is the number of processors used, *T1 *the execution time of one single processor and *Tp *the execution time of MOLA with *p *processors.

### Preparation steps

**Figure 1:**
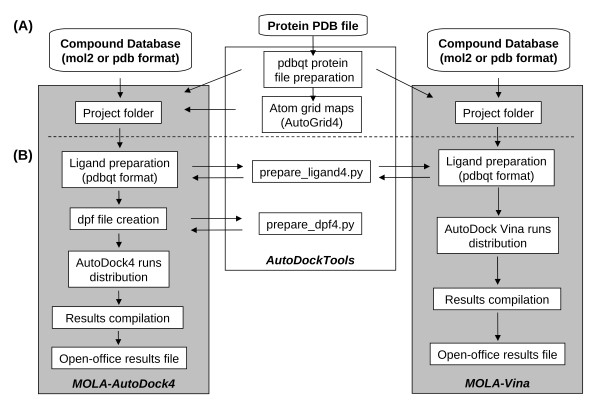
**Work-flow of MOLA software**. When a virtual screening project is initiated, MOLA software performs automatically several tasks for both AutoDock4 and Vina (grey rectangles). Some tasks rely on third-party tools that are automatically called when need (white rectangle). This scheme also presents: (A) the steps need to prepare the input files before launching MOLA and (B) the steps automated by MOLA.

MOLA was designed to work alongside ADT and integrates some scripts from this software package (figure 1). Before using MOLA for virtual screening some input files preparation steps are necessary (figure 1A) and are described in detail on MOLA tutorial. When using AutoDock4 the input files needed for the project include: the protein input file (in pdbqt format), ligand input files (pdb, mol or pdbqt file format) and atom grid maps. We use ADT to prepare protein input file as well as for calculating the atom grid maps. The original protein structure files are downloaded from the PDB (Protein Data Bank) website. ADT is available in the customized operating system with a "runAdt" command. Computing atom grid maps is done using AutoGrid4 [3]. With Vina the process is the same except that calculating the atom grid maps is no longer necessary as Vina calculates them automatically.

### Using MOLA for Virtual Screening

Once all input files are prepared MOLA is started by double clicking MOLA-AD4.sh or MOLA-Vina.sh files (figure 1B), placed on the/home/user folder. A GUI will appear and the user will be asked to input information about the project. For MOLA-AD4 the user is asked to select the project folder, the protein folder (where the protein and the atom grid maps are placed), the ligands folder, the active site coordinates and to choose several AutoDock4 parameters: number of runs, population size and energy evaluations. With MOLA-Vina the user is asked to select the project folder, the ligands folder, and to input: the grid center coordinates, grid area dimensions, the active site coordinates and exhaustiveness parameter (see tutorial for more information). MOLA was written as a shell script and the GUI interface prepared using the Xdialog package [19].

Once these parameters are chosen everything else is automated. If the compounds used are in pdb or mol2 file format, MOLA-AD4 and MOLA-Vina then automatically prepare pdbqt files for each compound using prepare_ligand4.py, a Python script from ADT. If the compound files are already on pdbqt format this step is skipped. When using MOLA-AD4, dpf files for each compound are created (dpf is need for docking with AutoDock4) using the Python script "prepare_dpf4.py" available on ADT. At this point MOLA's parallel algorithm is initiated and distributes 2 AutoDock4 jobs for each node. Every 3 seconds MOLA scans each node process list for the 2 initial AutoDock4 jobs, if 0 or 1 is found MOLA assumes AutoDock4 jobs have ended and initiates 2 or 1 AutoDock4 jobs, respectively. This algorithm was optimized for single and dual-core computers in order to make take full advantage of their processing power but MOLA script can be easily changed to be used with quad or octo-core computers (see tutorial). When using MOLA-Vina the algorithm works in the same manner except that the dpf file creation step is not necessary. Also only 1 run is sent for each node as Vina is already optimized for multi-core computers.

When MOLA finishes a results-AD4.csv or results-VINA.csv file is created and automatically opened as an open-office spreadsheet with the results presented in table format for easy interpretation and handling. The ligands are ranked by lowest binding energy and by distance to the active site. MOLA calculates this distance (in Angstroms) by measuring the distance from the active site center point, given by the user, to the docked ligand center mass point. The center mass point is calculated using the pdb_centermass.py script from pdb-tools suite [20]. All output files are recorded on a results folder created inside the project folder.

## Results and Discussion

In order to test both versions of MOLA we used 4 well known receptor-ligand complexes: retinol binding protein-retinol (1RBP), HIV-1 protease-XK263 (1HVR), trypsin-benzamidine (3PTB) and streptavidine-biotin (1STP). As the test compound database we used the first 500 from the NCI diversity set II [7]. All 4 protein receptors were prepared using ADT and saved on the project folder.

For this test both AutoDock4 and Vina treat the protein receptors as rigid molecules and ligands as flexible molecules. We defined the grid center as the geometrical center of the bound ligand in the receptor-ligand complex experimental structure. The search space (grid volume) was calculated considering the ligand dimension and increasing it by 15 Å on each dimension. This insures that the search space is large enough for the ligand to rotate in [3]. Using MOLA's GUI we selected the following AutoDock4 parameters: 50 LGA (Lamarckian Genetic algorithm) runs, a population size of 100 and 250000 energy evaluations. With Vina the default parameters were used except that we used the single threaded execution parameter (CPU = 1) to perform the MOLA serial test (table 1).

**Table 1:** Execution times of MOLA using 1 (serial), 2, 6 and 10 processors (parallel).

			Execution time (minutes)			
			
PDB code	Receptor-Ligand Complex		Serial	Parallel		
			
			1	2	6	10
1RBP	Retinol binding protein-retinol	Vina	1110	593	216	132
		
		AutoDock4	2650	1385	499	310

1HVR	HIV-1 protease-XK263	Vina	1034	536	195	120
		
		AutoDock4	2646	1366	484	306

3PTB	Trypsin-benzamidine	Vina	1108	583	211	129
		
		AutoDock4	2627	1367	495	302

1STP	Streptavidine-biotin	Vina	1025	535	193	119
		
		AutoDock4	2646	1369	493	305

To evaluate the performance of MOLA we used clusters for virtual screening with 2, 5 and 10 CPUs (parallel) and compared the performance against the same task on just 1 CPU (serial) (table 1). We achieved an average throughput of 237 ligands/CPU/day for AutoDock4 and 575 ligands/CPU/day for Vina. In our test Vina was about 2.5 times faster than AutoDock4 (table 1). This different in performance increases if we use more computer demanding AutoDock4 parameters.

During the virtual screening tests one computer was used as master node and the remaining computers were used as slave nodes. Of special concern was the possibility that the master node couldn't manage all the data traffic going out to and coming in from the slave nodes, but the system worked flawlessly on all the tests. To date we tested MOLA flawlessly with up to 20 processors (10 dual-core computers) and don't anticipate problems when adding more computers to the cluster. Still, for larger clusters, attention to the master node is advised to insure it can handle all In/Out files operations. Also it's important to note that the master node computer should be the fastest computer available.

**Figure 2:**
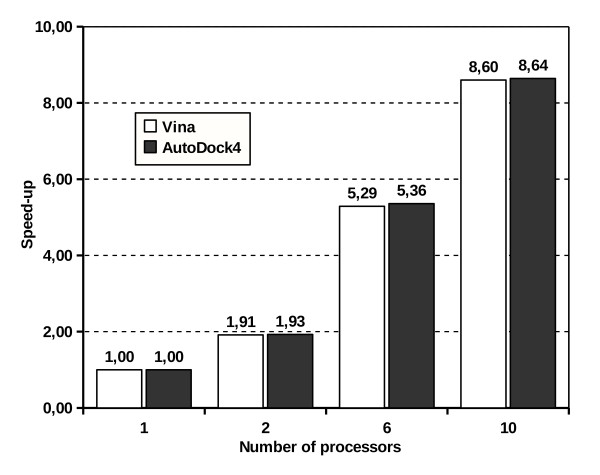
**Speed-up obtained with MOLA using different sizes of computer clusters**. The values of speed-up were obtained dividing the execution time of the parallel algorithm of MOLA (with 2, 6 and 10 processor clusters) by the execution time of the sequential algorithm of MOLA with 1 processor. The execution times were calculated averaging the values of the 4 proteins used to test MOLA using AutoDock4 (dark grey) and Vina (white).

MOLA's parallel algorithm was designed to scan for AutoDock4 or Vina jobs launched on each node every 3 seconds. This methodology assures that computers with very different processing speeds can be added to the cluster and made to work at their own pace. Slower computers only receive more jobs after finishing the last ones. With this approach a small loss in cluster performance was expected as, for each job launched, a lag phase of up to 3 seconds occurs. Still for MOLA we observed a good speed-up reaching, on a 10 CPU cluster (maximum potential speed-up of 10×), 8,64× using AutoDock4 and 8,60× using Vina (figure 2). The small performance loss observed is acceptable when we consider that the trade of is being able to add a diverse set of computers to our cluster.

As most dedicated clusters, the computers used for this test have similar configurations, a situation considered important for clusters stability. Still MOLA was designed to be used with computers with different processing speeds and characteristics. Extensive testing with all computers available to us was made (data not shown). To date we still haven't found a computer that couldn't be added to the cluster.

**Figure 3:**
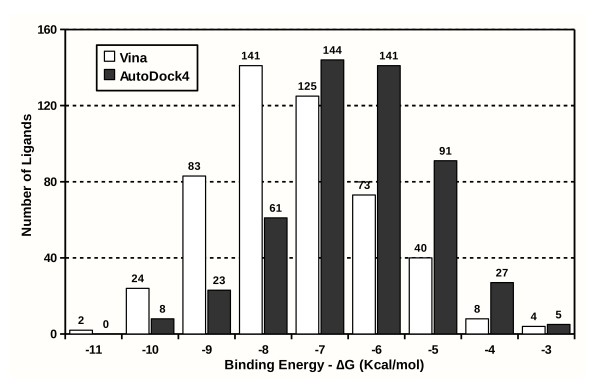
**Virtual screening results using MOLA with Retinol binding protein as protein target**. We used Retinol binding protein as example protein target and the first 500 compounds of the NCI diversity set II database as ligands. The results for AutoDock4 (dark grey) and Vina (white) are presented as the number of ligands on each of the binding energy Kcal/mol) intervals presented. The bar-graph was prepared with open-office using the results-AD4.csv and results-VINA.csv files automatically opened in the end of the virtual screening experiment.

At the end of a project MOLA places the result files of all ligands in a results folder and integrates the results creating a results-AD4.csv or results-VINA.csv. These files are automatically opened as an open-office software spreadsheet, with the ligands ranked by binding energy and distance to the active site. The spreadsheet table format makes the results much easier to store, interpret and analyse. This can be seen in figure 3 that shows the docking results for Retinol binding protein presented in a bar graph produced with open-office were we can see the ligands distribution according to binding energy. The characteristic bell shape distribution of the ligands is very similar when comparing both AutoDock4 and Vina. Still we observed a consistent shift to lower binding energy with Vina for all the 4 receptors used. MOLA also calculates the distance of the docked ligand to the active site and this is relevant as it gives you an immediate indication of how close the ligand virtually binds to the protein site of interest. Ligands with lower binding energy and smaller distance to the site of interest can be immediately highlighted as the most promising compounds. There is no need for further steps using third-party software, usually used to visually inspect the ligand-protein complex for each ligand.

## Conclusions

In this work we present MOLA an easy-to-use software tool that performs parallel virtual screening of compound databases against protein receptors, using AutoDock4 or Vina as docking engines. MOLA automates several tasks including: ligand preparation, docking jobs distribution and results analysis. The design of MOLA's parallel algorithm was thought-out so that the cluster remains stable even when using computers with different speeds and characteristics. MOLA uses as operating system a customized Live-CD GNU/Linux operating system that connects all the computers and assembles the cluster. The customized operating system uses RAM memory insuring that the installed operating system and the hard-disk drive in not used. All results are automatically recorded on a USB-flash drive or on the hard-disk drive if using VirtualBox. From cluster assembling to docking result analysis, MOLA is very easy to use and the tutorial present a step-by-step approach that ensures a smooth virtual screening experience. MOLA is not intended for users that have access to large dedicated computer clusters rather was developed for non-experienced users that want to develop small virtual screening projects by using non-dedicated multi-platform computers clusters.

## Availability and Requirements

Project name: MOLA

Project homepage: http://www.esa.ipb.pt/~ruiabreu/mola

Operating System: Platform independent (Linux/Unix, Windows, MAC)

Programming language: Shell Scripting and Xdialog GUI tool-kit

Other requirements: No requirements

License: GNU GPL

Any restrictions to use by non-academics: None

## Competing interests

The authors declare that they have no competing interests.

## Authors' contributions

RMVA was involved in the design and implementation of the software and the manuscript preparation. HF was also involved in the design and implementation of the software and also in testing and benchmarking the software. MJRPQ and ICFRF were involved in the supervision of the project, manuscript preparation and intellectual guidance. All authors read and approved the final manuscript.

## Supplementary Material

Additional file 1 **Tutorial for MOLA**. This tutorial presents a complete step-by-step guide to use MOLA, including cluster assembly and virtual screening with MOLA using AutoDock4 or Vina. Also the preparation steps before using MOLA and the possibility of using VirtualBox for booting is explained in detail. Click here for file
