# Supplementary material for: MOLA: a bootable, self-configuring system for virtual screening using AutoDock4/Vina on computer clusters
Source: J Cheminform. 2010 Oct 28;2:10. doi: 10.1186/1758-2946-2-10 (PMC2987878; doi:10.1186/1758-2946-2-10)
Supplement: Additional file 1 — Tutorial for MOLA. This tutorial presents a complete step-by-step guide to use MOLA, including cluster assembly and virtual screening with MOLA using AutoDock4 or Vina. Also the preparation steps before using MOLA and the possibility of using VirtualBox for booting is explained in detail. [file 1758-2946-2-10-S1.PDF]

Tutorial for MOLA

June 2010

Authors: Hugo Froufe and Rui Abreu

MOLA is an easy-to-use software for Virtual Screening (VS) in non-dedicated computer clustera using AutoDock4/Vina. Several tasks needed for AutoDock4/Vina are automated with MOLA including: ligand preparation, AutoDock4/Vina jobs distribution, result analysis and ligand ranking. A Graphical User Interface (GUI) is used for easy selection of parameters and easy handling of input/output files generated in a VS project. MOLA is integrated in a Live-CD GNU/Linux Live Distribution designed to boot from the CD on any available computer without ever using the computer hard-disk drive. When a VS project is finished the computers can be restored to the original operating system by simply removing the CD and restarting.

PART 1 of this tutorial addresses the computer cluster assembly while PART 2 and 3 explains the use of MOLA with AutoDock4 and Vina, respectively.

To use MOLA you need to download MOLA.iso file and burn it to a CD then just restart you master node computer from the CD following the instructions on this tutorial. An alternative way is using VirtualBox as a virtual machine. In this case you boot directly from the MOLA.iso file without the need to burn a CD (see APPENDIX 2 of this tutorial).

You can use MOLA on multiple-core workstations or you can network multiple computers together to assemble a cluster. The computer slave nodes boot by PXE, using the master node as the server. More recent computers (about less than 5 years old) usually have the option for PXE booting so this step is automatic. However for older computers with no PXE option you can use gPXE.

This tutorial consists of 3 PARTS and 2 APPENDIXS:

PART 1. Cluster assembly. Needed for both AutoDock4 and Vina …..3

PART 2. Virtual Screening with MOLA using AutoDock4..9

PART 3. Virtual Screening with MOLA using Vina..17

APPENDIX 1 Preparation steps using AutoDockTools (ADT) before using MOLA.................22

APPENDIX 2. Using VirtualBox instead of booting from the the CD.......................................33

Material need:

-1 multi-core computer or 2+ computers connected via ethernet cables. When using 2 computers you can connect them directly with a cross-wire ethernet cable (normal ethernet cables won't work). For 3 or more computers you need all computers linked using normal ethernet cables via a switch.

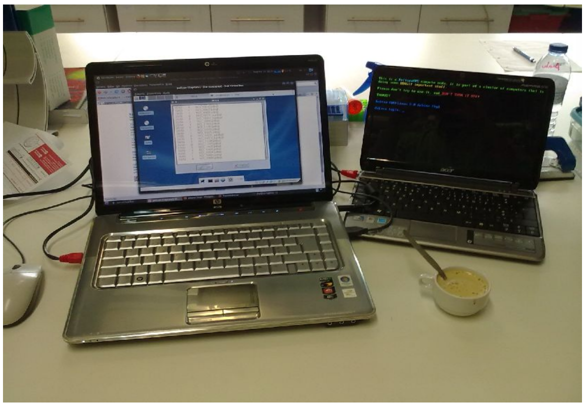

2 computers cluster

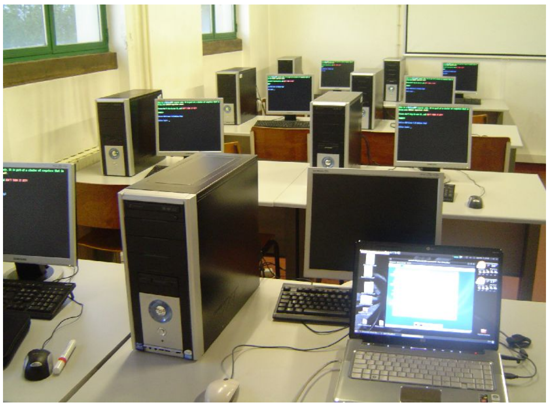

8 computers cluster

-1 Good sized USB-flash drive (2 GB or more) or a USB-hard disk drive for storage (using VirtualBox as alternative you can make a direct link to your hard-disk drive and use it for storage). The images show computer cluster examples with the master node using VirtualBox on a Ubuntu Linux Host.

- MOLA CD (download iso file from www.esa.ipb.pt/~ruiabreu/mola).

Files need for this tutorial (and present on the \home\user\tutorial-files folder):
- 1RBP.pdbqt example protein file.
- 1RBP protein atom grid maps for AutoDock4.
- NCI diversity set2 compound dataset in pdbqt format.

This tutorial assumes that you are using the tutorial files to test MOLA. We use Retinol Binding Protein (RBP) as test protein. The tutorial was written in a way that, if you follow all steps, you can perform a VS project using RBP without ever leaving the customized Live-CD Linux operating system and with no prior experience.

To prepare the files need for VS with your own protein some steps are need. For this we have included on the customized Live-CD operating system the software package AutoDockTools (ADT). ADT is a software that allows the user to prepare the files need for docking with AutoDock4 and Vina. A detailed tutorial for preparing the files used on this tutorial (from the original 1RBP.pdb file) is presented on APPENDIX 1.

PART 1- Cluster assembly

1. To use MOLA first you need to download the MOLA.iso file from: http://www.esa.ipb.pt/~ruiabreu/mola, and burn it to a CD. PART 1 is partially based on the original tutorial for Pelican HPC tutorial with some adaptations necessary to use MOLA. For more detailed information you can find the tutorial for Pelican HPC here: http://econpapers.repec.org/paper/aubautbar/749.08.htm. Remember you can also use VirtualBox instead of using a CD (see Appendix2 first).

2. Place the CD in the computer CD device of your master node computer and turn it on. For your master computer node you should use the faster computer with more RAM memory. When you boot up, you'll see something like the following screen. By default most computers boot first from the CD. If this is not your case you need to go to your boot setup (usually pressing F8 during booting) and select the CD device as your primary booting device, then just restart the computer.

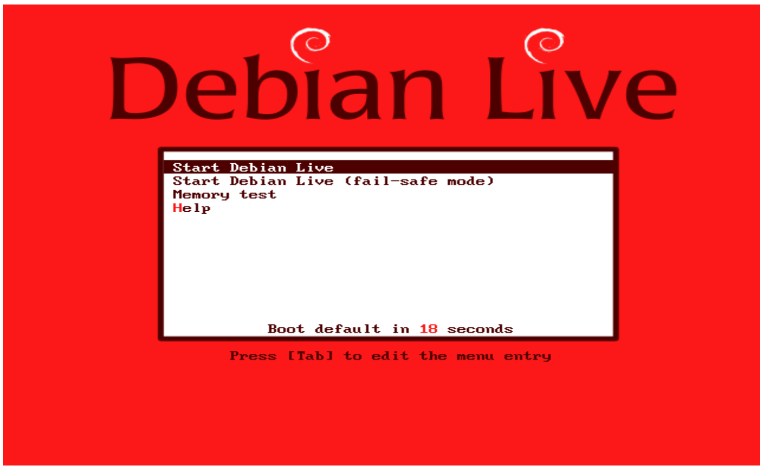

3. During booting eventually you will see the next screen. To use MOLA you do not need this feature so just click 'OK'. This screen gives you the opportunity to use a permanent storage device for the '/home/user' folder but for MOLA we strongly recommend not using this option, just press "OK"!
"This feature is provided as a convenience for advanced users but it's impossible to test this feature on all possible hardware configurations, so NO GUARANTEES ARE MADE THAT IT WILL NOT DESTROY YOUR HARD DISK. Back up your data before trying anything but the default" (quote from Pelican HPC tutorial).

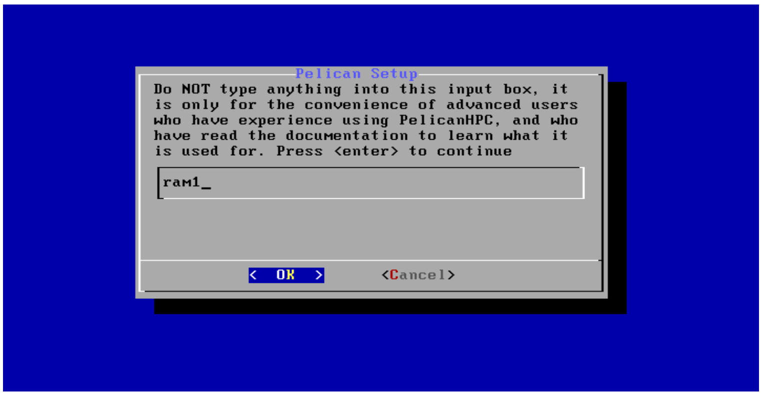

4. Next, you will see this screen. Just choose ‘yes’.

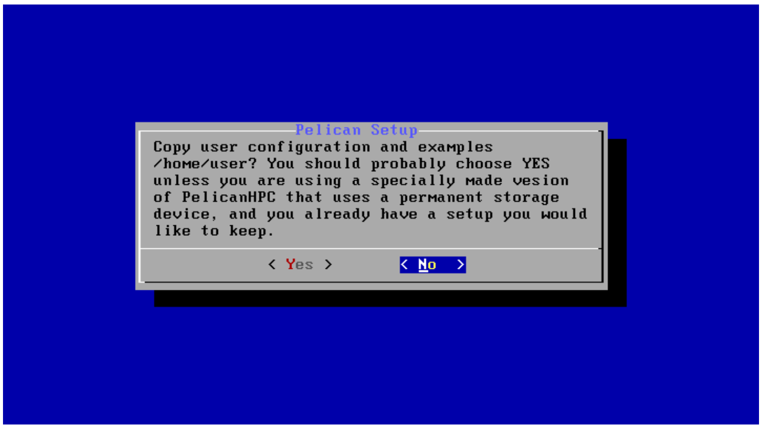

5. Next, you are prompted to change the default password. You should backspace to remove the default, then type in a new password and click ‘OK’.

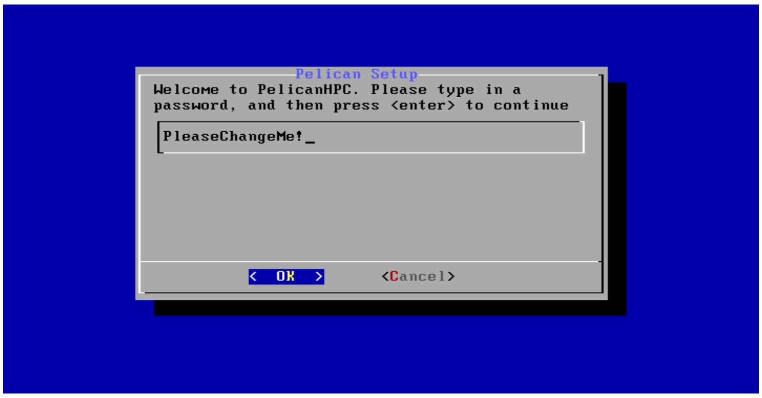

6. Finally, you are all booted up and the login prompt appears. Enter the username ‘user’ and then the password you created on step 5.

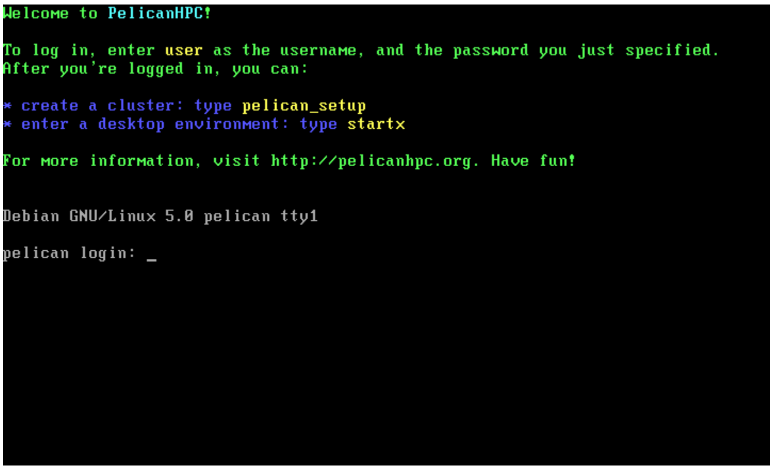

7. Now start the Xfce graphical interface by typing 'startx'.

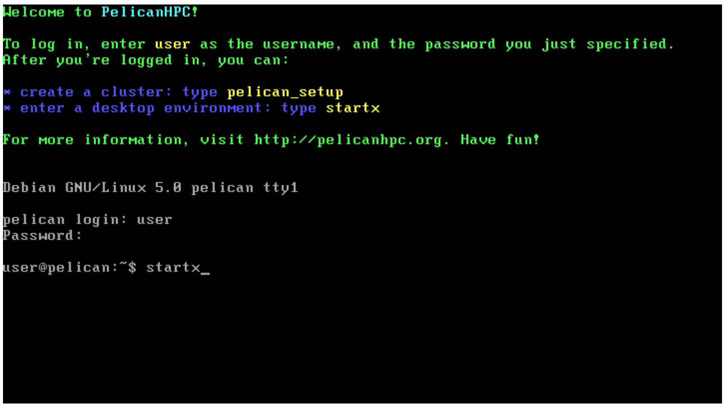

8. Now you should see the Xfce graphical interface and you are ready to assemble the cluster. Click on the Console as indicated by the red arrow to open a command-line interface.

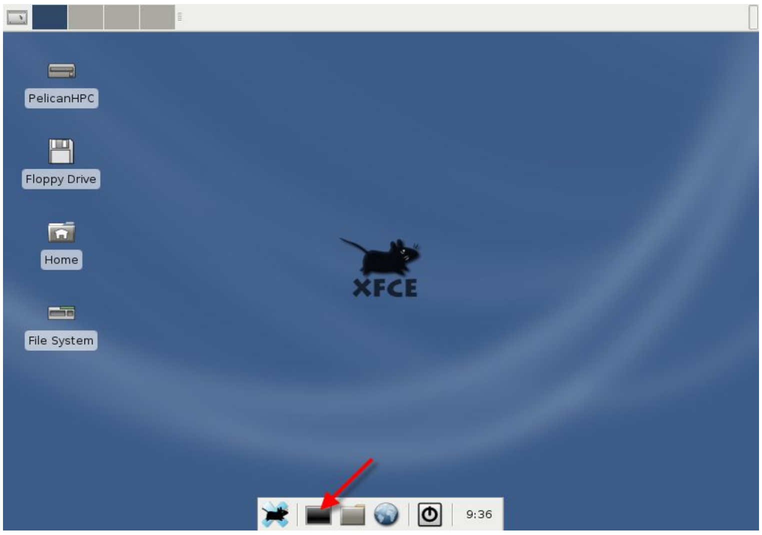

9. To set up the cluster, type 'pelican_setup' and click 'Enter'.

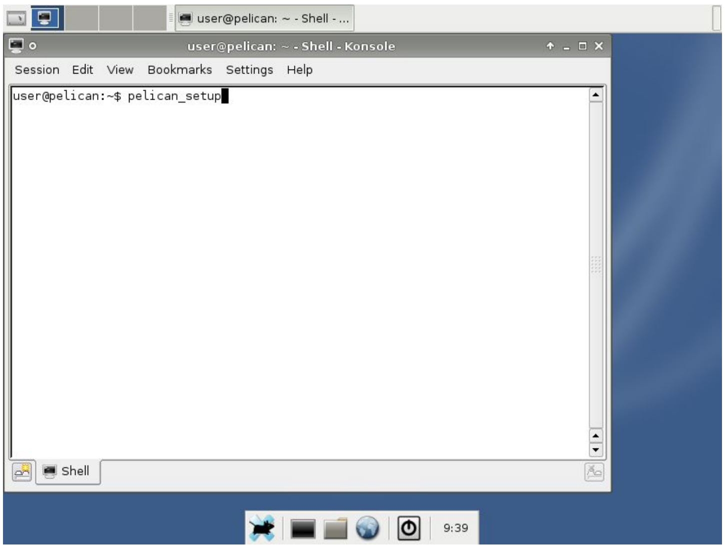

10. If you have more than one network device you will be asked to choose the net device, a screen will appear to select the device you will use (usually the first option). Next, we see the following screen. Please read the warning in the screen carefully. You should make sure that the network device used for the cluster is isolated from all networks except the cluster. When you see the following screen, choose ‘Yes’.

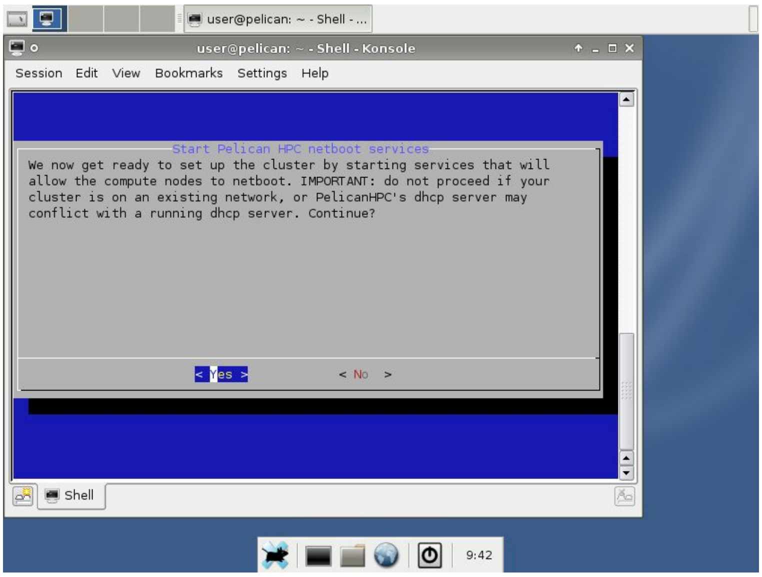

11. Next you will see this screen. Choose ‘Yes’ and click ‘Enter’.

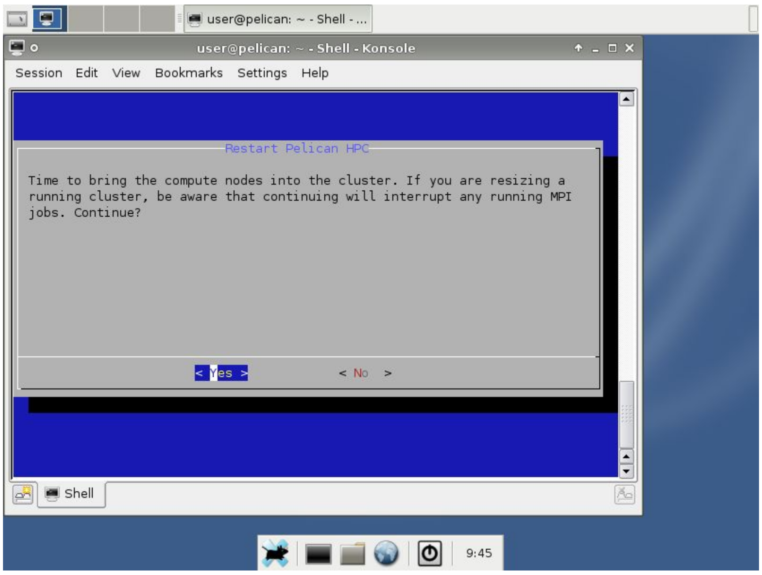

12. When you see the next screen, go turn on the other computer slave nodes.

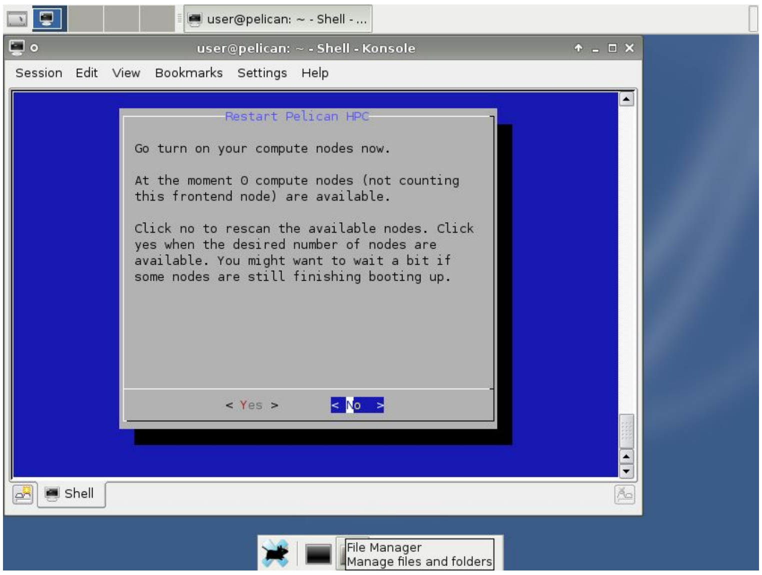

When a compute node starts to net-boot using PXE you'll see the next screen. If this screen doesn't appear the computer may be too old to have PXE, in this case see NOTE1 at the end of this PART.

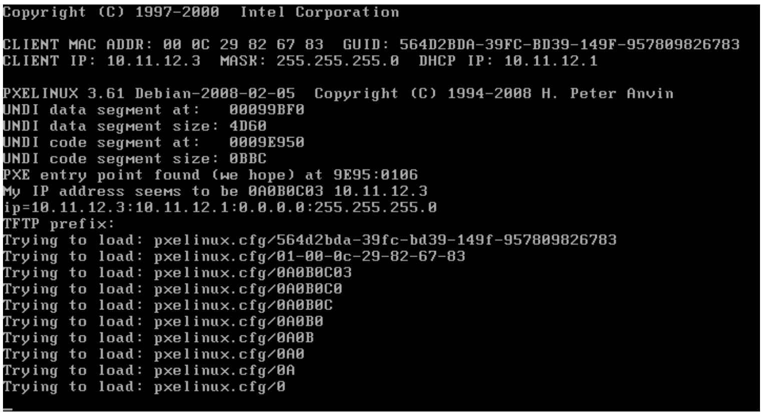

When a compute node is done booting, you'll see this:

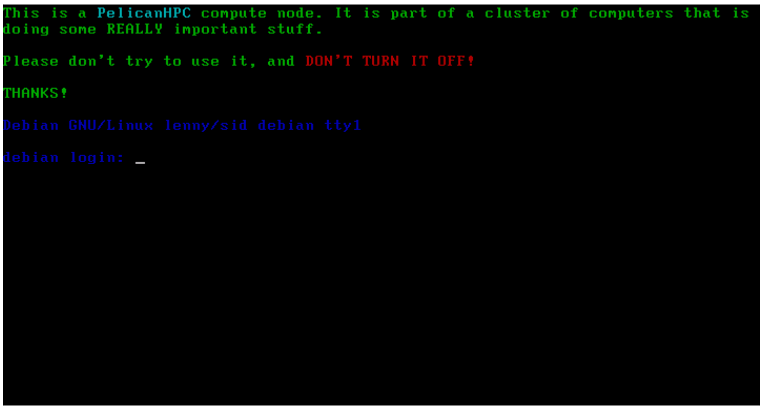

13. Click ‘No’ to rescan and now you should see this. Once a node has booted up, the count goes up.

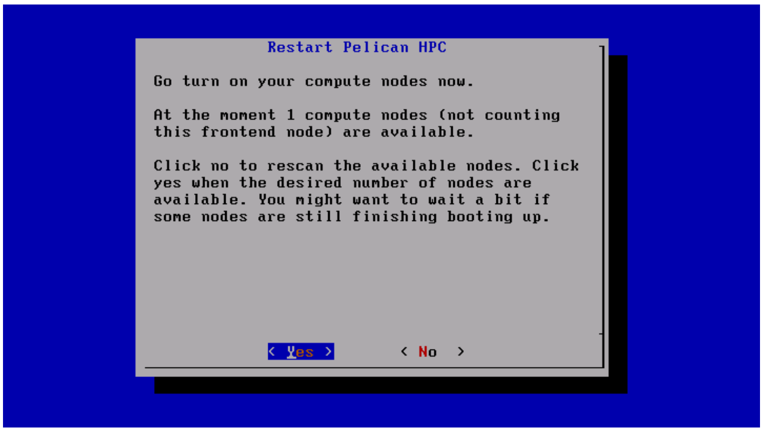

14. Keep hitting ‘No’ until all of your compute nodes have booted up. Once you click yes, you'll see something like the next screen, depending on how many nodes you have. You can check if the cluster is assembled and how many nodes are connected by typing the command ‘lamnodes’ on the Console. In our example we use only 2 computers (a master and a slave) connected using a crosswire ethernet cable.

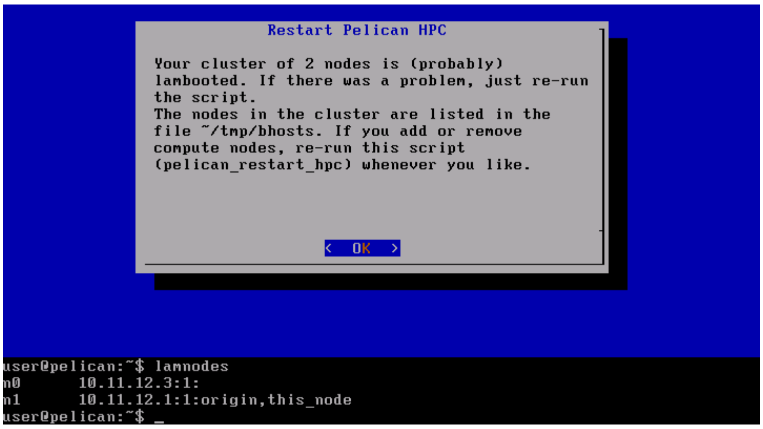

OK, that's it, the cluster is ready to use!

NOTE 1. Remember that, for older computers ,you may not have the PXE option. In this case you can use gPXE for booting. You can get it on this site http://rom-o-matic.net/gpxe/gpxe-git/gpxe.git/contrib/rom-o-matic/ . Just burn the gPXE.iso to a CD and boot the slave node computer from the CD as we have done with the master node computer.

PART 2. Virtual Screening with MOLA using AutoDock4.

We are now going to use MOLA with AutoDock4 as docking engine. We use as protein target the Retinol Binding Protein (RBP) and as ligands the NCI diversity set II compound dataset. For this tutorial we use the first 50 of 1364 compounds of the NCI diversity set II compound dataset that is available with the MOLA Linux distribution.

The files need for this tutorial are all on the '/home/user/tutorial-1RBP' folder and, for the purpose of testing MOLA in this tutorial, we are going to do everything from this folder. However remember that as the files are placed on transient RAM memory, if the computers are turned off, all the files on the '/home/user' folder disappear. For a real-life Virtual Screening project you should do everything from a USB device. Just connect the USB device and it will appear on a '/media/disk' folder with an icon on the desktop.

There are 2 versions of MOLA available: one for use with AutoDock4 (MOLA-AD4v1.0.sh) and other for Vina (MOLA-VINAv1.0.sh). They are separate as you may want to use just one of the docking engines, still you can start both MOLA version at the same time that they will handle the jobs seamlessly and at the same time.

1. Click the 'Home' icon on the Desktop and the '/home/user/' folder opens (blue arrow). This folder contains the files need for VS with MOLA. You can see the 2 versions of MOLA (red arrows). The tutorial files needed to test MOLA are on the 'tutorial-1RBP' folder (orange arrow). You can also find this tutorial as 'tutorial-MOLA.pdf'. There is a 'tools' folder were some tools used by MOLA are located. Also you can find a 'share' folder created to link to my hard-disk drive but this folder is only used with when we are assembling the cluster with VirtualBox software (see Appendix 2).

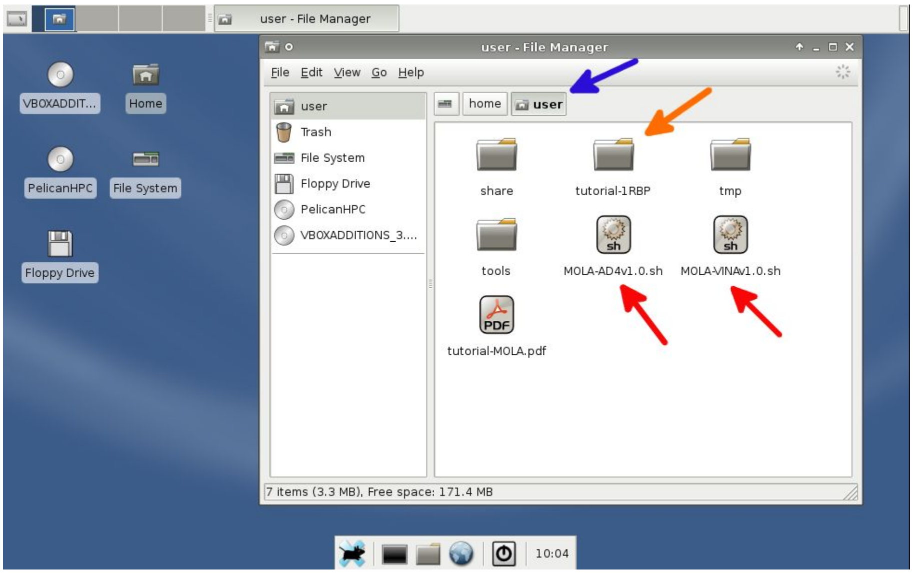

Important! If one of the computers from the cluster is quad-core or octo-core, you can take full advantage of their processing power. Open MOLA-AD4V1.0.sh with Kate text processing software (type on the Console this command line 'kate MOLA-AD4v1.0.sh') and read line 208 for instructions.

2. Now you are going to run the 'MOLA-AD4v1.0.sh' executable located on '/home/user'. Double click on 'MOLA-AD4-v1.0.sh' (brown arrow)*. The MOLA windows opens. You are now going to be asked for the location of the input files for MOLA. First select a PROJECT folder (pink arrow)**. For this tutorial we use the '/home/user/tutorial-1RBP' folder as PROJECT folder (green arrows). In this folder you have all the files you need for your VS project. Also it's in this folder that all the result files are recorded on a 'results-AD4' folder. Click 'OK'.

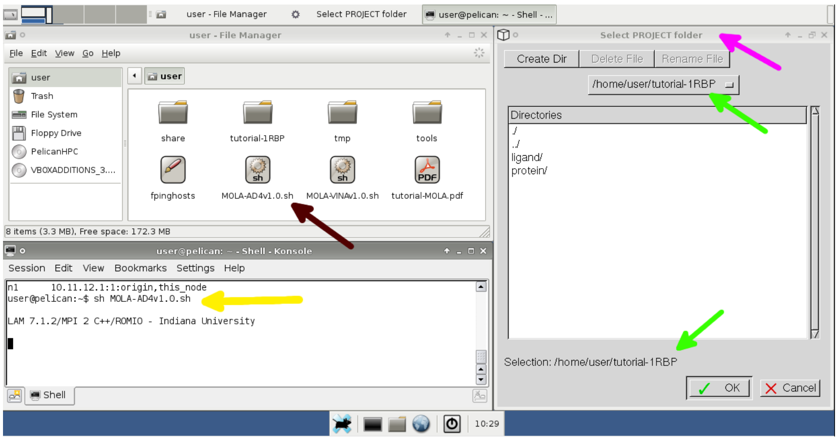

* As an alternative open a Console (black box on the bottom) and write this command line 'sh MOLA-AD4v1.0.sh' (yellow arrow). Starting from a Terminal allows you to see the context messages but that extra information although not essential for using MOLA. ** Remember that, for a real VS project, you are advised to locate the PROJECT folder on a USB device for proper physical storage of the results. Just plug a USB-flash drive that will appear as '/media/disk' folder. Then just place the tutorial-1RPB folder on it and select the '/media/disk/tutorial-1RBP' as PROJECT folder, the rest of this tutorial is the same. This insures the results are recorded in a physical storage device as the docking jobs of each compound finish.

3. Now select the PROTEIN folder (blue arrow). On this folder there should be the atom grid map files needed for docking with AutoDock4 (see APPENDIX 1 on how to prepare the maps). For this tutorial we will select the '/home/user/tutorial-1RBP/protein' folder (red arrows) were the tutorial atom grid maps files are placed.

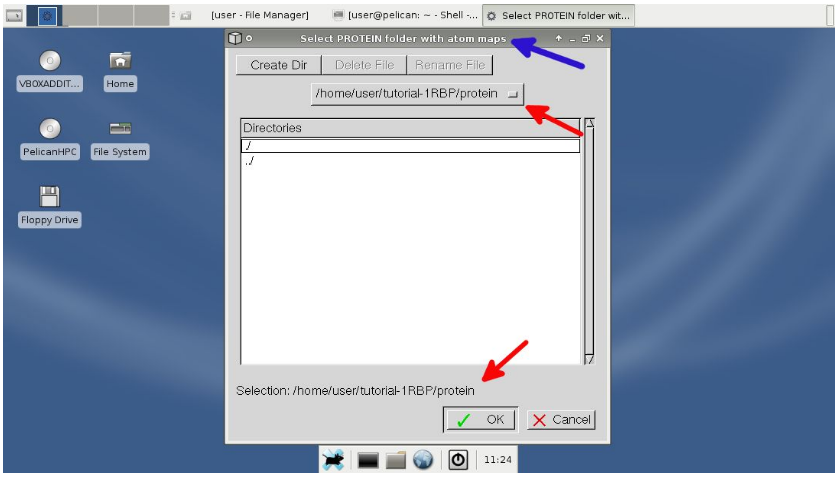

4. Now select the PROTEIN file in PDBQT format (orange arrow). For this tutorial select the '1RPB_receptor.pdbqt' also located on the '/home/user/tutorial-1RBP/protein' folder (yellow arrows).

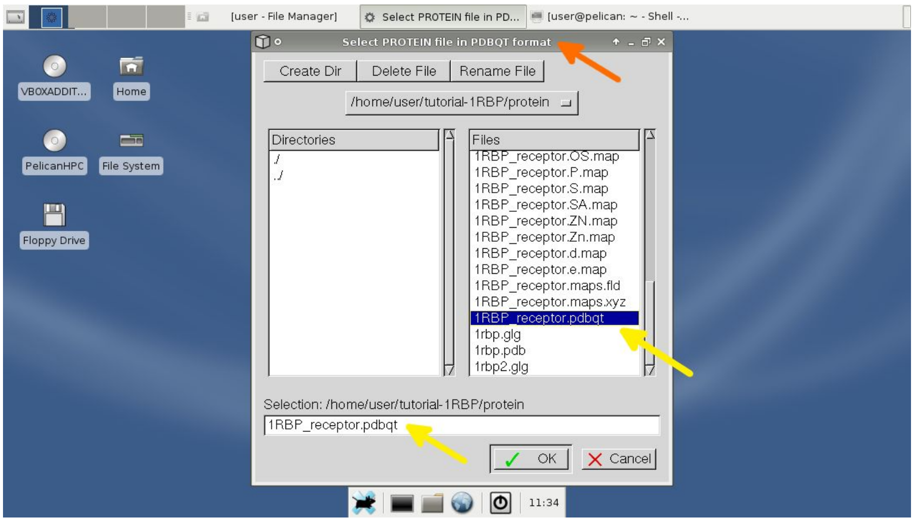

5. Now select the LIGAND folder (green arrow). On this folder compound files you want to use with MOLA must be present. For this tutorial select the '/home/user/tutorial-1RBP/ligand/tutorial-ligand' folder (pink arrows) as the ligands are placed here. On this folder you have 50 compounds from the 1364 that compose the NCI diversity set II compound dataset, already in PDBQT format. If you want to use the complete 1364 dataset you have them available at '/home/user/tutorial-1RBP/ligand/NCI_diversitySet2' folder, but if you select this folder it will take a while! The allowed formats are: PDBQT, PDB and MOL2. PDBQT is the file format required for AutoDock4. If the ligands are in PDB or MOL2 MOLA will automatically convert them to PDBQT.

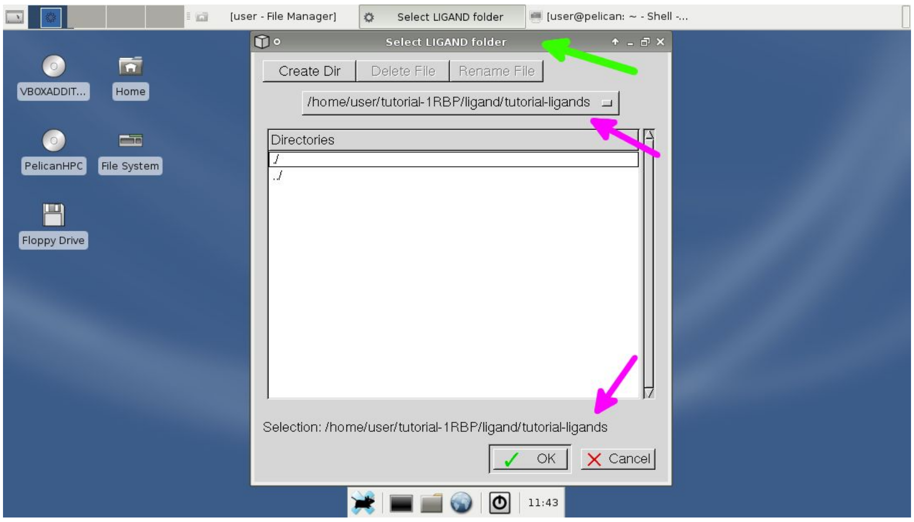

6. Now select AutoDock4 parameters. Some values are given for reference purpose, use the values you want having in mind that the higher the parameters values the more reliable are the results but the longer it takes. We will use the parameters: 250000 as number of evaluations parameter (black arrow), 150 as population number (blue arrow) and 50 as number of runs (red arrow). Click 'OK'.

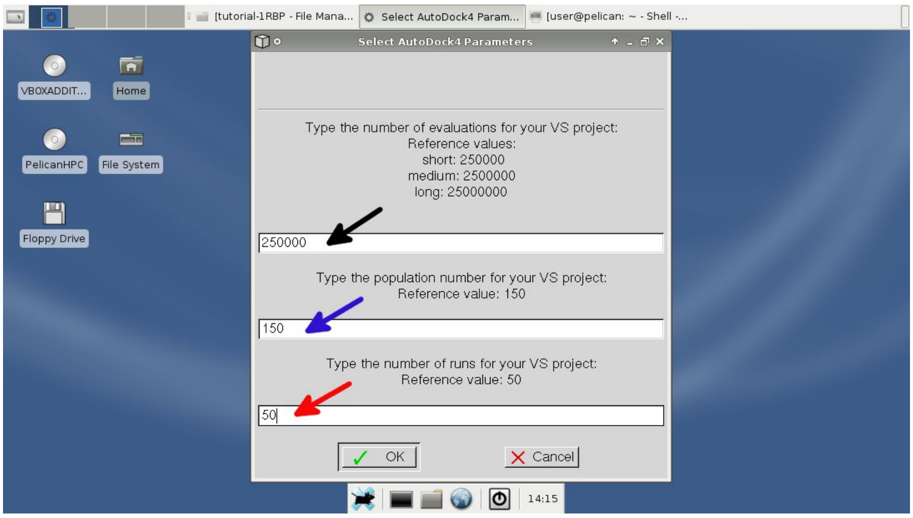

7. You can now enter the XYZ coordinates of the ACTIVE SITE (or bound experimental ligand coordinates). We use 33.2 18.9 and 51.2 for XYZ coordinates respectively, these values are the approximate center of the ACTIVE SITE of RBP protein structure we are using and also the grid centre (see APPENDIX 1). Click 'OK'.

These values are used to calculate the distance, in Angstroms, of each virtually docked ligand to the coordinates of a position of interest. Usually this position is the ACTIVE SITE were we want to know if the ligand binds to it (or at least docks in the proximity). It can also be the position of a ligand experimentally bound to the protein structure we are using. The smaller the distance the closer the ligand binds to the ACTIVE SITE and thus the more relevant the ligand as possible protein modulator.

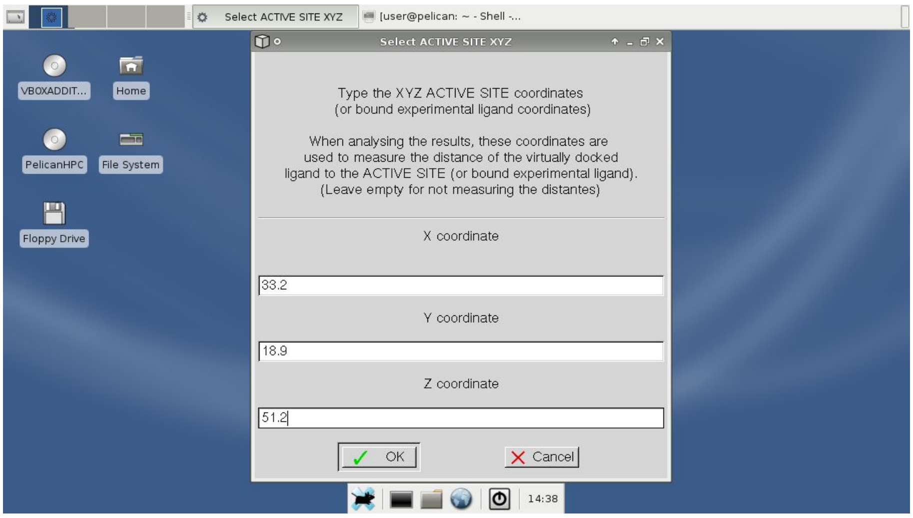

8. Finally an information box appears were you can review all the input data. If everything is OK click 'Yes'.

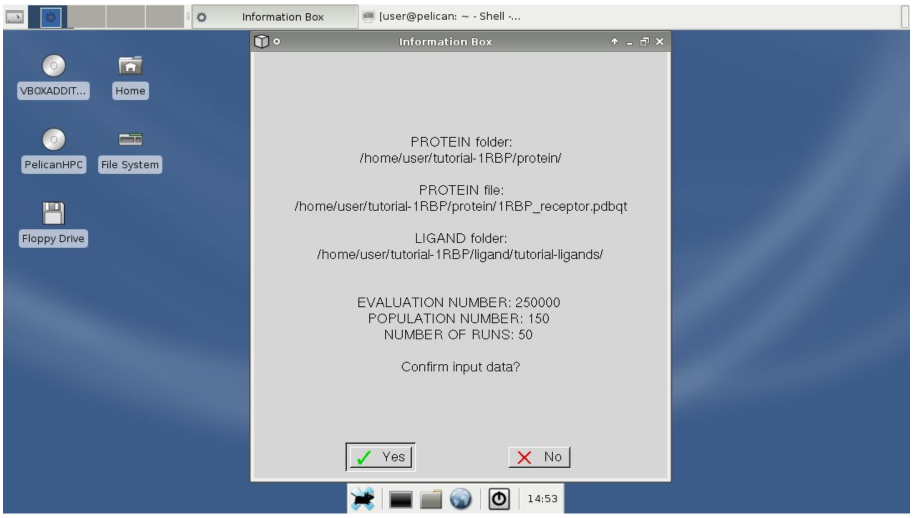

9. NOW you see a MOLA monitoring window were you can observe all the steps MOLA automatically performs on a VS project. First MOLA prepares the ligand files (orange arrow), if the files are in PDB or MOL2 format they are converted to PDBQT format in this tutorial the files are already in PDBQT format so MOLA continues to the next step. Then MOLA creates a DPF file for each ligand, this step is needed for AutoDock4 to run. Notice that the first ligand is 1RBP_ligand.pdbqt (see APPENDIX 1 on how to create it), that corresponds to retinol the experimentally bound ligand of our protein. Retinol will work as a control compound.

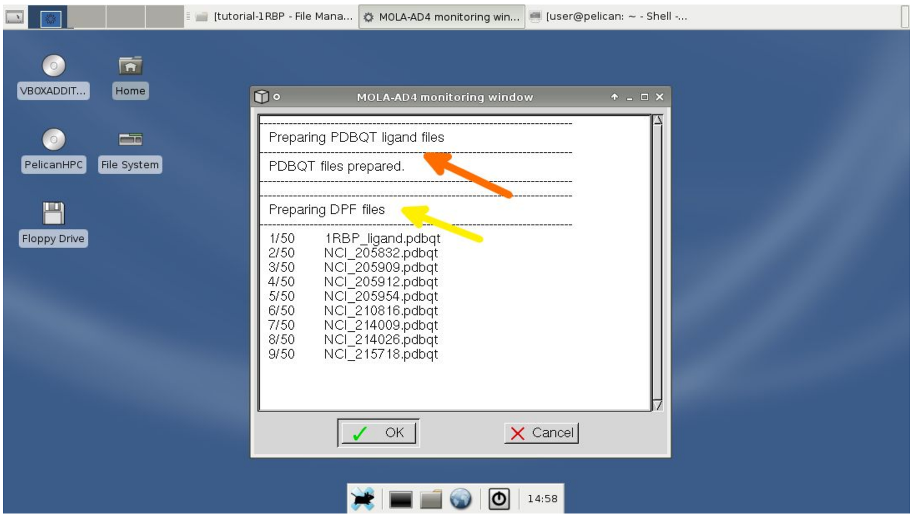

10. With all DPF files created MOLA starts launching AutoDock4 jobs to the computers nodes of your cluster (green arrow). You can monitor this process on MOLA window were you can see: the jobs already launched, the node on where the job was launched and the ligand being docked. In this tutorial we are using a 2 computer cluster so we have nodes 0 and 1 (the highest number is always the master node, in this case node 1). MOLA sends 2 AutoDock4 jobs to each computer. When 1 jobs finishes MOLA automatically sends another until all jobs are launched.

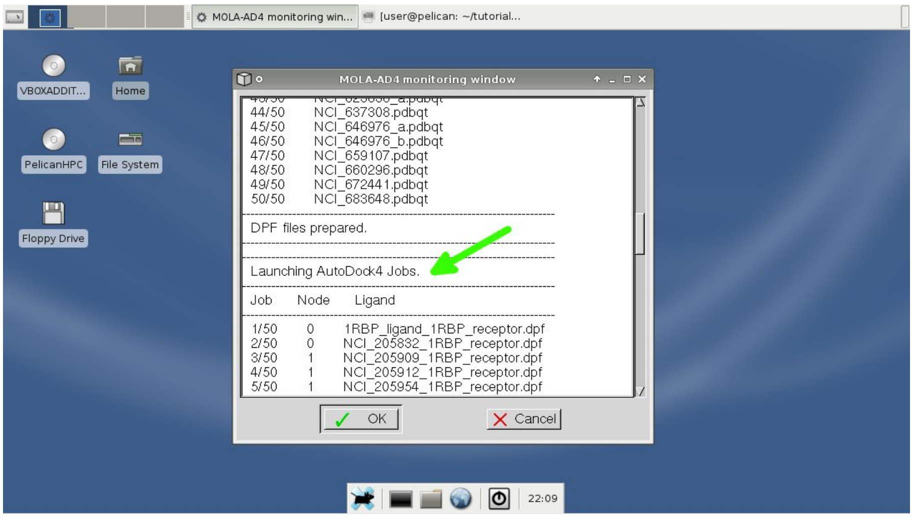

11. When all jobs are finished a message appear on the MOLA window (pink arrow). The biggest cluster we tested so far had 10 dual-core computers with a total of 20 processors. You can also just use MOLA as a multi-core workstation. This is useful when you only have one computer and don't want to manually start each AutoDock4 job.

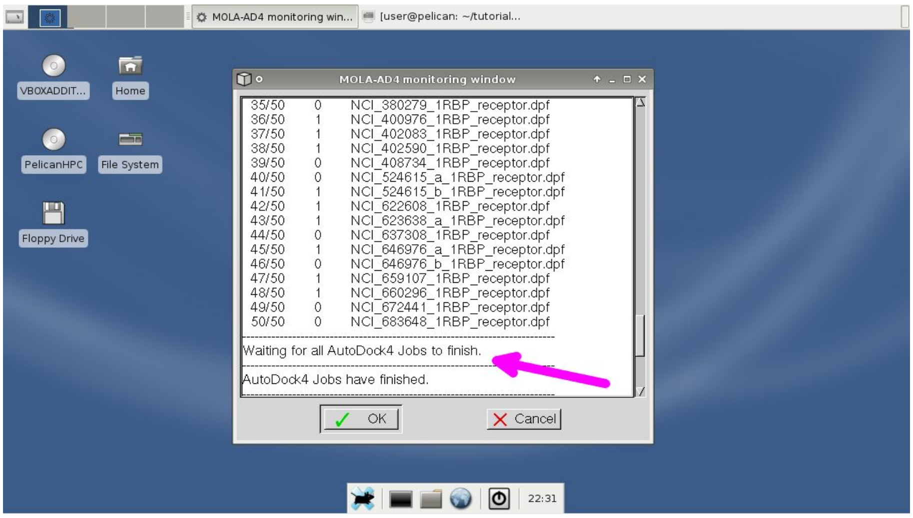

12. MOLA them starts the result analysis step (black arrow). This step can take some time depending on the number of compounds used because several calculations need to be done (about 2 to 5 seconds per ligand). In the end an open-office spreadsheet is opened with the results presented in table format for easy interpretation and handling. Before opening the open-office spreadsheet an Text Import window appear (red arrow). Be sure to tick the Space option (blue arrow). Then click 'OK'.

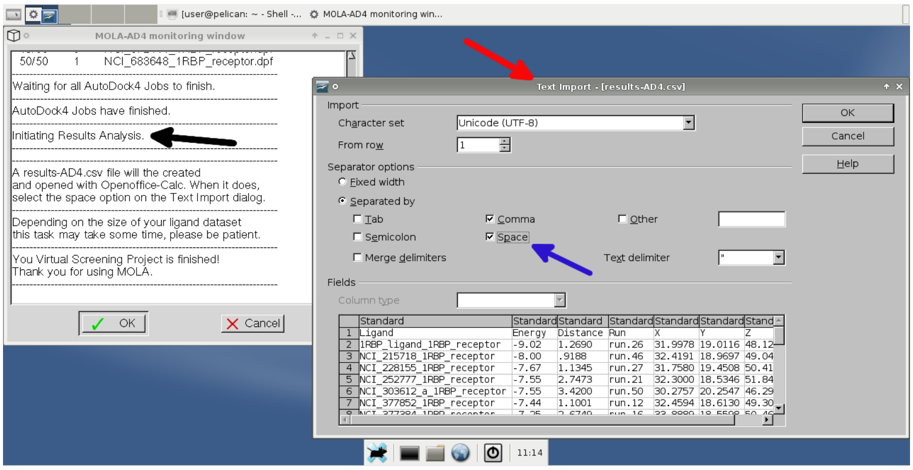

13. The open-office spreadsheet opens with the name results-AD4.csv. On this result table you can find several columns with different information:

column A: Ligand - ligand and receptor name

column B: Energy - lowest energy of binding - ΔG (Kcal/mol)

column C: Distance -The distance between the center mass of the virtually docked ligand with the lowest energy of binding and the ACTIVE SITE (left blank if no coordinates were given).

column D: RUN - AutoDock4 run corresponding to the lower energy value.

column EFG: XYZ coordinates – space coordinates of the virtually docked ligand with the lowest energy of binding.

The results are sorted according to the compound with lowest energy of binding. Remember we used Retinol as control. You can see that it's Retinol the first compound in the list with the lowest energy of binding, with the center mass located just 1.27 Angstroms from the selected ACTIVE SITE. The distance value is designed to give an immediate indication if the ligand virtually binds close to the ACTIVE SITE of interest. The higher the value the further away from the site of interest.

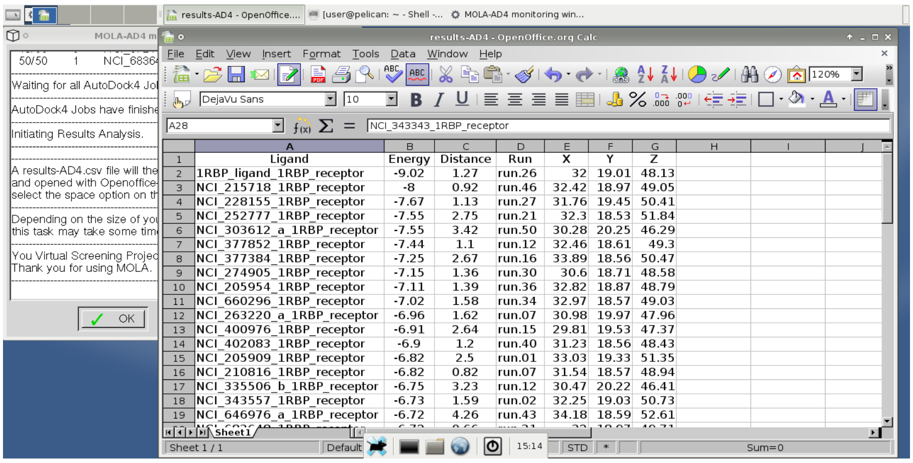

14. The results-AD4.csv (yellow arrow) file is recorded on a results-AD4 folder (orange arrow) in the PROJECT folder ('/home/user/tutorial-1RBP/results-AD4' in this tutorial). With the information presented on the results-AD4.csv file you can have an immediate overview on the quality of the VS results. Still for a visual inspection of the results a folder is created for each ligand on the results folder (green arrow).

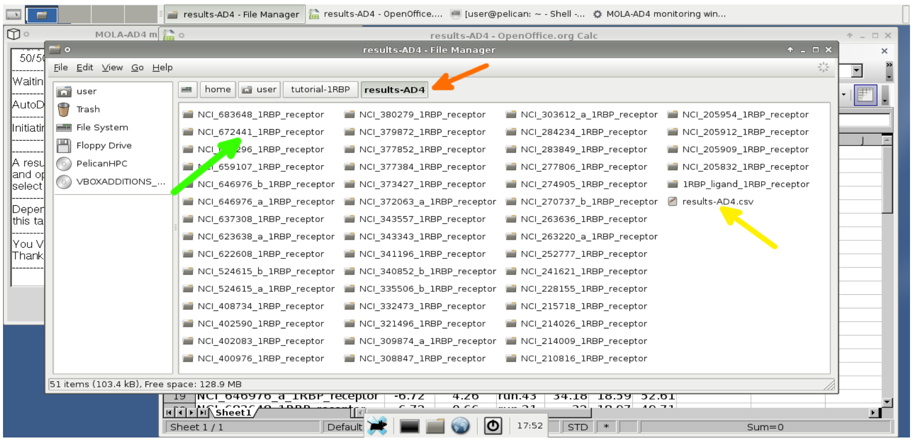

15. On each folder you have a DLG (docking log) file (brown arrow) that you can open with ADT to inspect all the docked ligand conformations. Also you can find, in separate PDB files, all the docked ligand conformations of each AutoDock4 run (black arrow) in this tutorial we selected 50 runs. These files are useful if you want of open the conformations in a different software like, per example, Pymol. Just check the run with the lowest energy of binding on the run column of the results-AD4.csv file.

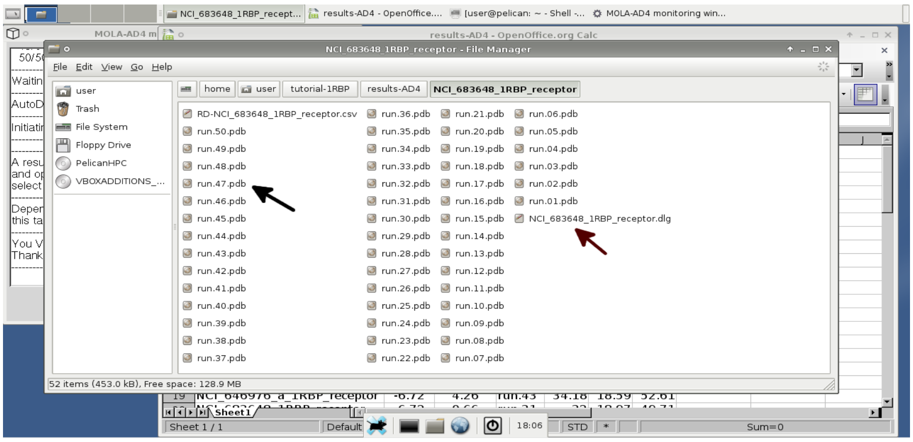

In many VS projects the 'results-AD4.csv' file may be all the results you need and you may delete the rest of the results files as they occupy a considerably amount of disk space (about 300 kilobyte on average per ligand). Remember that, in the results-AD4.csv' file, you have the “how strong it binds” information (Energy column) and the “were it binds” information (Distance column)!

PART 3. Virtual Screening with MOLA using Vina.

Using MOLA with Vina uses that same approach as using MOLA with AutoDock4. Still Vina and AutoDock4 work in different ways so some changes were made in MOLA-VINA. However both MOLA versions can be launched at the same time as they will will work seamlessly. The tutorial files used will be the same, the difference is that the result files using MOLA-VINA will be recorded on a results-VINA folder on the PROJECT folder.

1. Click the 'Home' icon on the Desktop and the '/home/user/' folder opens (blue arrow). This folder contains the files need for VS with MOLA using VINA. The tutorial files needed are on the 'tutorial-1RBP' folder (orange arrow). Now you are going to run the 'MOLA-VINAv1.0.sh' executable located on '/home/user'. Double click on 'MOLA-AD4-v1.0.sh' (brown arrow). The MOLA windows opens. You are now going to be asked for the location of the input files for MOLA. First select a PROJECT folder (pink arrow). For this tutorial we use the '/home/user/tutorial-1RBP' folder as PROJECT folder (green arrows). Click 'OK'.

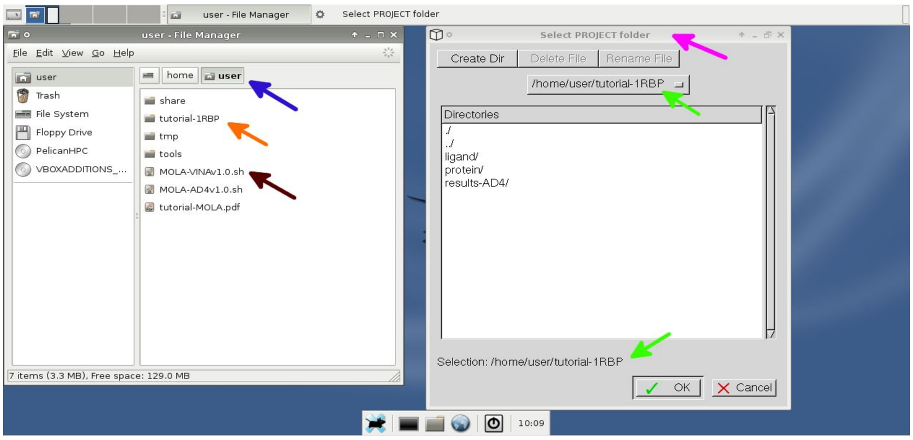

2. Now select the PROTEIN file in PDBQT format (red arrow). For this tutorial select the '1RPB_receptor.pdbqt' located on the '/home/user/tutorial-1RBP/protein' folder (yellow arrows). Notice that the atom grid maps used with AutoDock4 are no longer needed.

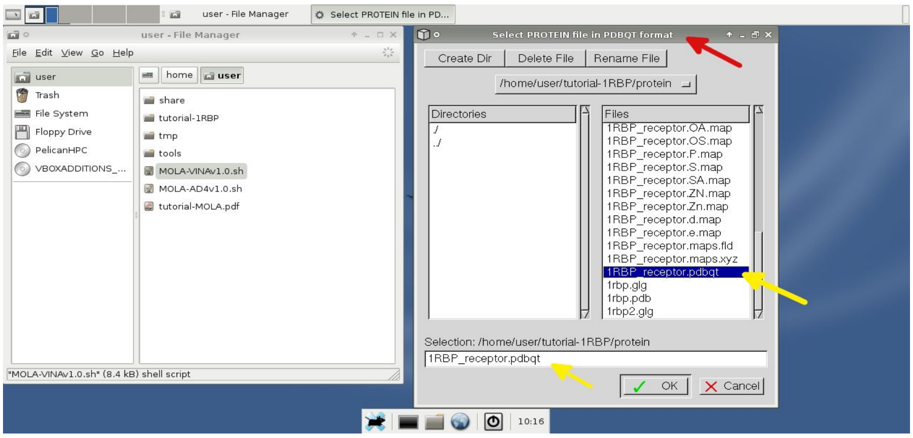

3. Now select the LIGAND folder (brown arrow). We use the same compound files used with MOLA using AutoDock4. For this tutorial the ligands used for testing are on the '/home/user/tutorial-1RBP/ligand/tutorial-ligands' folder (black arrows). On this folder we have 50 compounds from the 1364 that compose the NCI diversity set II compound dataset in PDBQT format. Click 'OK'.

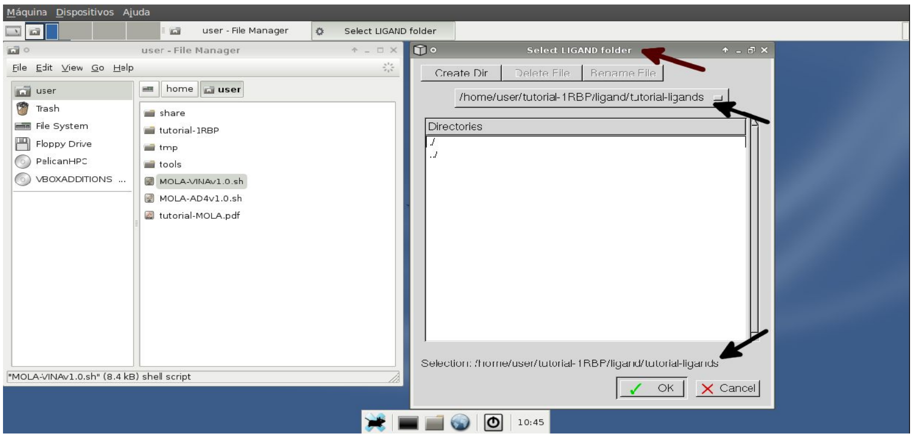

4. Now we insert VINA Parameters needed to use MOLA. For VINA you need to define 'search space' by inserting the center of the grid (XYZ coordinates) and the size of each dimension of the grid (xyz dimensions). We will use for this tutorial: 33.2 18.9 and 51.2 for XYZ coordinates (blue arrow) and 30x30x30 Angstroms for the XYZ dimensions size (orange arrow). Please see APPENDIX 1 on how these values are calculated. Also a level of 'exhaustiveness' needs to be set, the higher the value the more thorough is the global search at the expense of more processing time. The default is 8 (if you erase 'exhaustiveness' VINA assumes 8) but we will use 2 to speed up the process (pink arrow). Click ‘OK’.

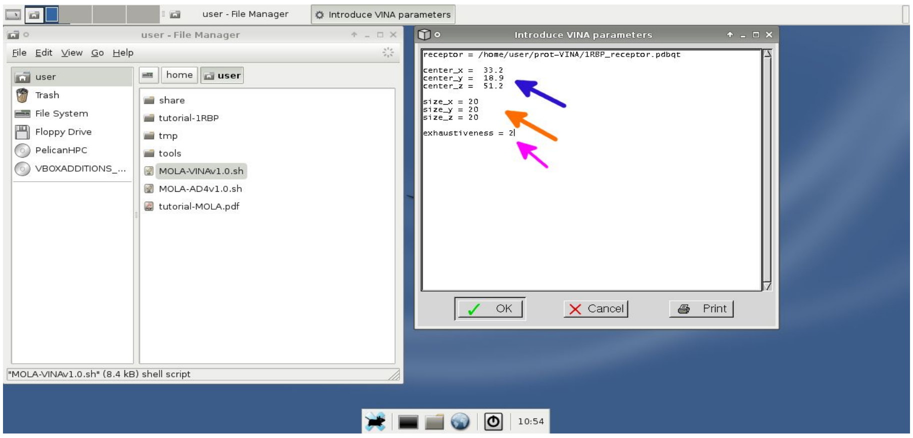

5. You can now give the XYZ coordinates of the ACTIVE SITE (or bound experimental ligand coordinates). We use the same values of the center grid coordinates as they coincide with the ACTIVE CENTER, in this tutorial use the values 33.2 18.9 and 51.2 for XYZ coordinates respectively (see APPENDIX 1). Click 'OK'.

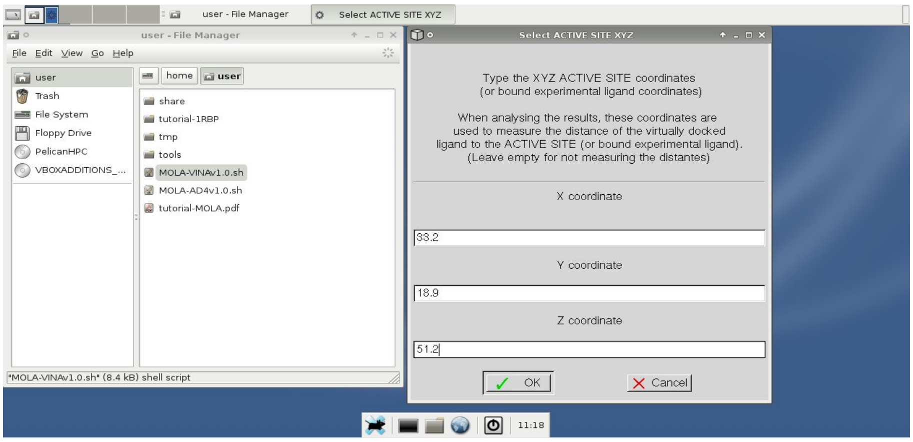

6. NOW you see a MOLA monitoring window were you can observe all steps MOLA automatically performs on a VS project. First MOLA prepares the ligand files (red arrow), if the files are in PDB or MOL2 format they are converted to PDBQT format, but in this tutorial the files are already in PDBQT format so MOLA continues to the next step. Then MOLA starts launching VINA jobs to the computers nodes of your cluster (yellow arrow). MOLA sends 1 VINA job to each computer node. When 1 jobs finishes MOLA automatically sends another until all jobs are launched. With dual-core computers VINA is able to use both processors so there is no need to send more than 1 job like with AutoDock4. Notice that MOLA sends more jobs to node 1 than to node 0, that is because node 1 has a faster processor and finishes a job faster. This shows that MOLA handles heterogeneous sets of computers efficiently and issue usually seen on dedicated computer clusters.

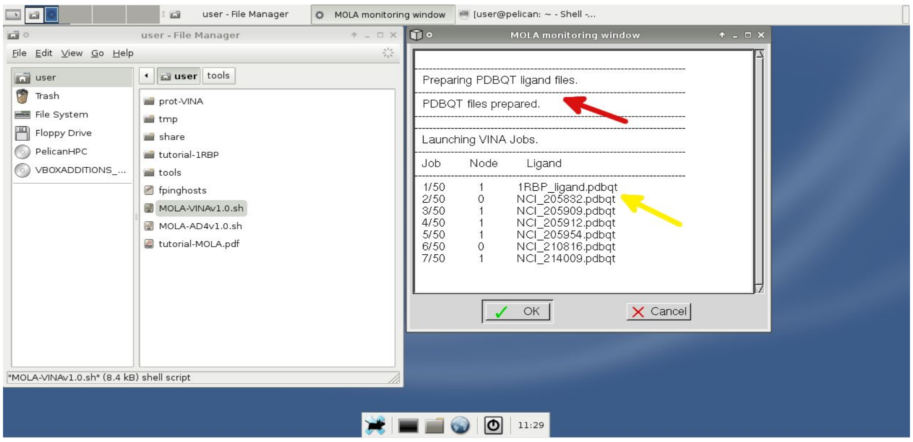

7. When all jobs are finished a message appear on the MOLA window (pink arrow). The biggest cluster we tested so far had 10 dual-core computers with a total of 20 processors (see picture X). You can also just use MOLA as a workstation (one computer cluster). This can be useful when you only have one computer and don't want to manually start each Vina job.

MOLA them starts the result analysis (blue arrow). This step can take some time depending on the number of compounds used because several calculations need to be done (about 2 to 5 seconds per ligand). In the end an open-office spreadsheet is opened with the results presented in table format for easy interpretation and handling. Before opening the open-office spreadsheet an 'Text Import' window appear (red arrow). Be sure to tick the Space option (black arrow). Then click 'OK'.

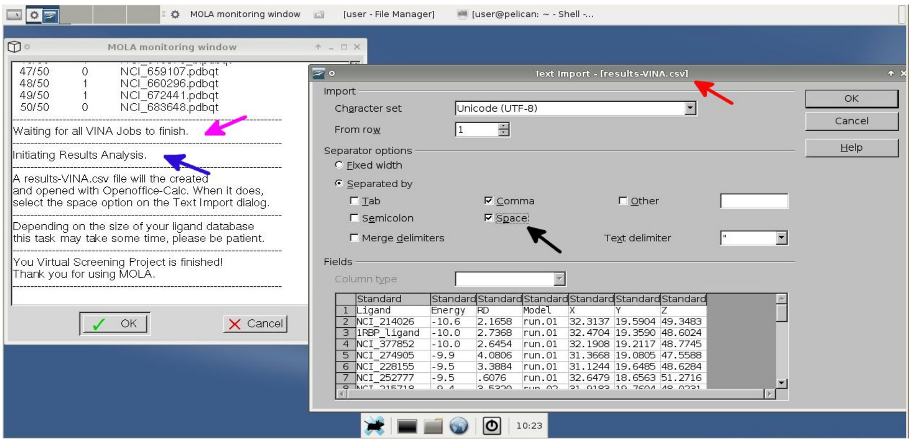

8. The format of the results with MOLA-Vina is the same as with MOLA-AD4, so please see information on step 13 of PART2. Retinol (1RBP_ligand) is expected to rank high and, as we can see, is ranked 2 in 50 compounds (highlighted).

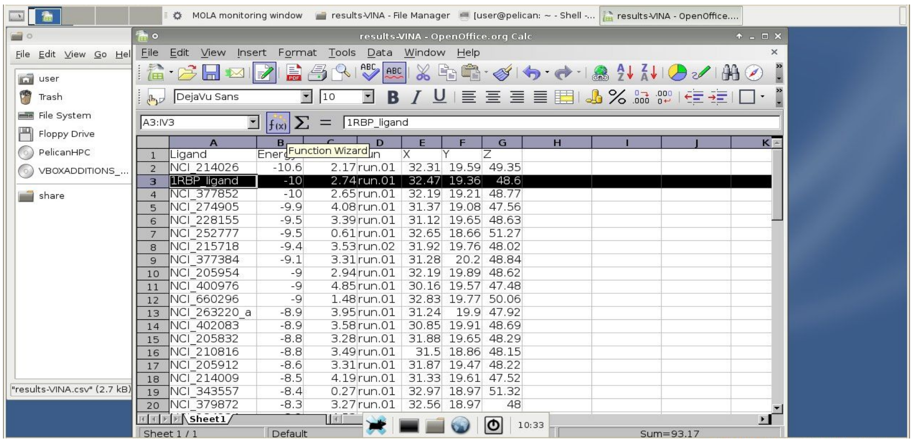

9. The open-office spreadsheet opens with the name results-VINA.csv (yellow arrow). This file that is recorded on a results-VINA folder (orange arrow) in the PROJECT folder. With the information presented on the results-VINA.csv file you can have an immediate overview on the quality of the VS results. Still for a visual inspection of the results a folder is created for each ligand on the results folder (green arrow).

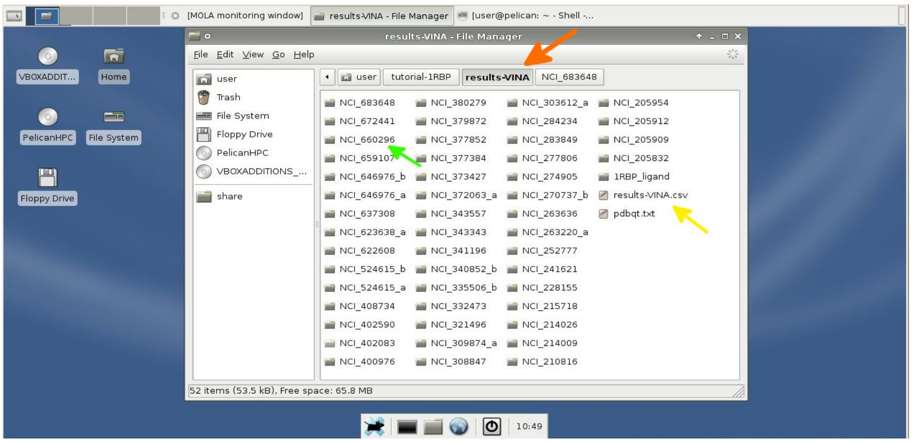

10. On each folder you have a out.X.pdbqt file (brown arrow) that you can open with ADT to inspect all the docked ligand conformations. Also you can find, in separate PDB files, all the docked ligand conformations of each VINA run (black arrow), for VINA the conformation with lowest binding energy is usually run1. These files are useful if you want of open these conformations in a different software like, per example, Pymol. Just check the run with the lowest energy of binding on the run column of result-AD4.csv file.

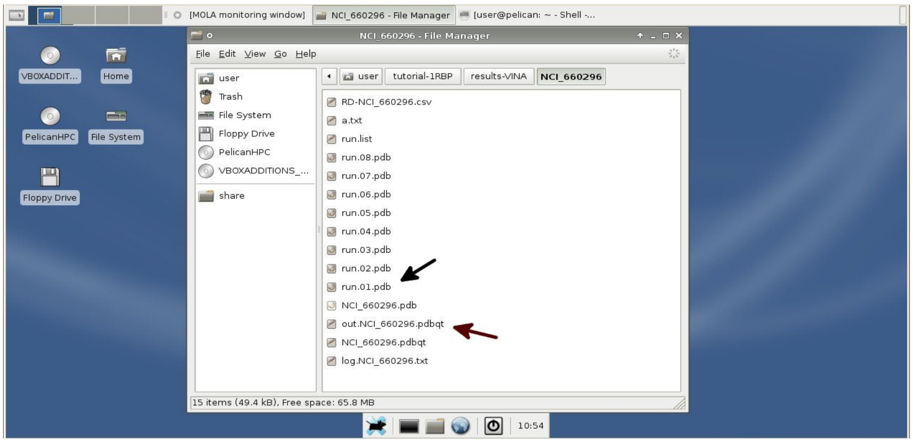

In many VS projects the 'results-AD4.csv' file may be all the results you need and you may delete the rest of the results files as they occupy a considerably amount of disk space (about 300 kilobyte on average per ligand). Remember that, in the results-AD4.csv' file, you have the “how strong it binds” information (Energy column) and the “were it binds” information (Distance column)!

We’ve reached the end of this tutorial!

Hope MOLA will we a useful tool in your VS projects!

APPENDIX 1. Preparation steps using AutoDockTools (ADT) before using MOLA

On this appendix we will learn how to prepare the input files need for MOLA. For this we will use AutoDockTools (ADT). For convenience, ADT is included on MOLA Live-CD distribution, however you can use ADT on any operating system of your choice. If you have experience with ADT you probably don't need appendix 1.

1. The files needed for this tutorial are placed on the PROTEIN '/home/user/tutorial-1RBP/protein/' folder. You will need the '1RBP.pdb' (red arrow) file and the 'atom-types.txt' file that are already of the PROTEIN folder.

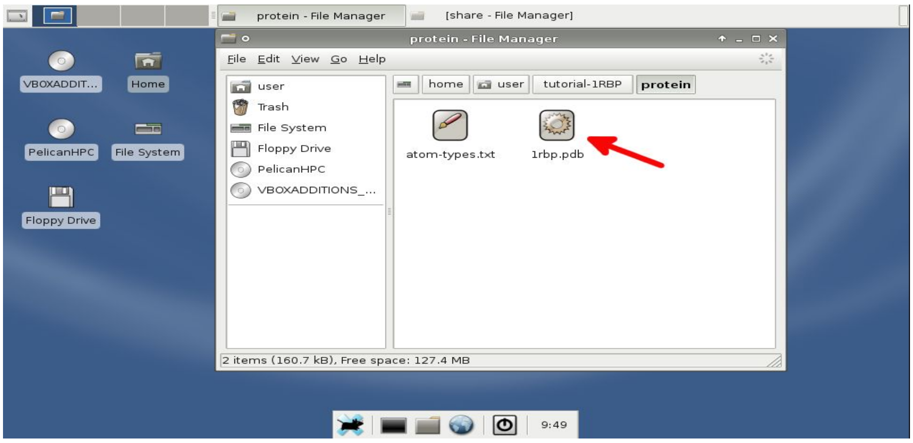

2. Left click on the 1RBP.pdb file, select 'Open with other application' and select the Kate program. Now you see the PDB file with the coordinates of all atoms. The RBP.pdb file contains the structure of the Retinol Binding Protein (RBP) in complex with the natural ligand Retinol. In this case the ligand must be removed from the PDB file before docking with AutoDock4 or Vina. Also waters and other non-protein compounds are removed. Highlighted we see Retinol atoms (indicated by the RTL abbreviation).

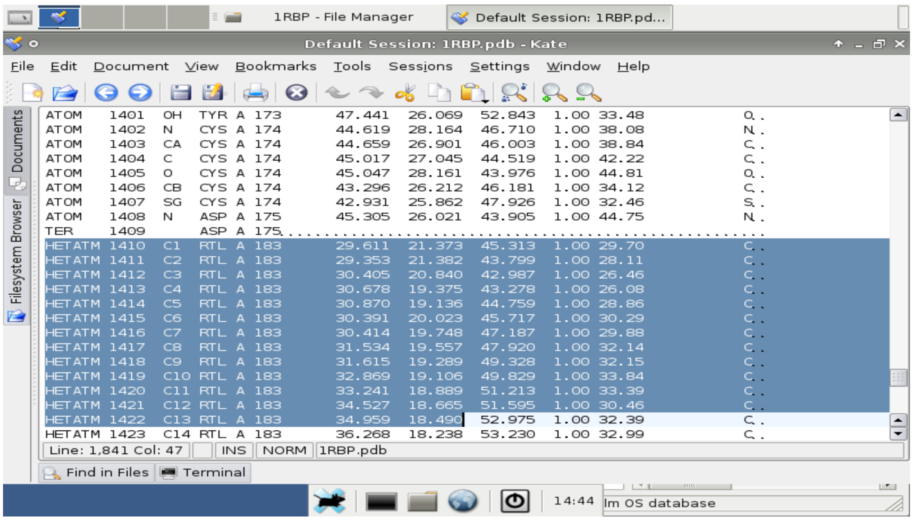

3. Select all lines starting with HETATOM and CONNECT and delete them. ATOM lines refer to atoms belonging to the protein, HETATOM lines refer to atoms that do not belong to the protein but are interacting with the protein (ligands, co-enzymes, waters, etc). After deleting the lines, the end of the file should be something like this:

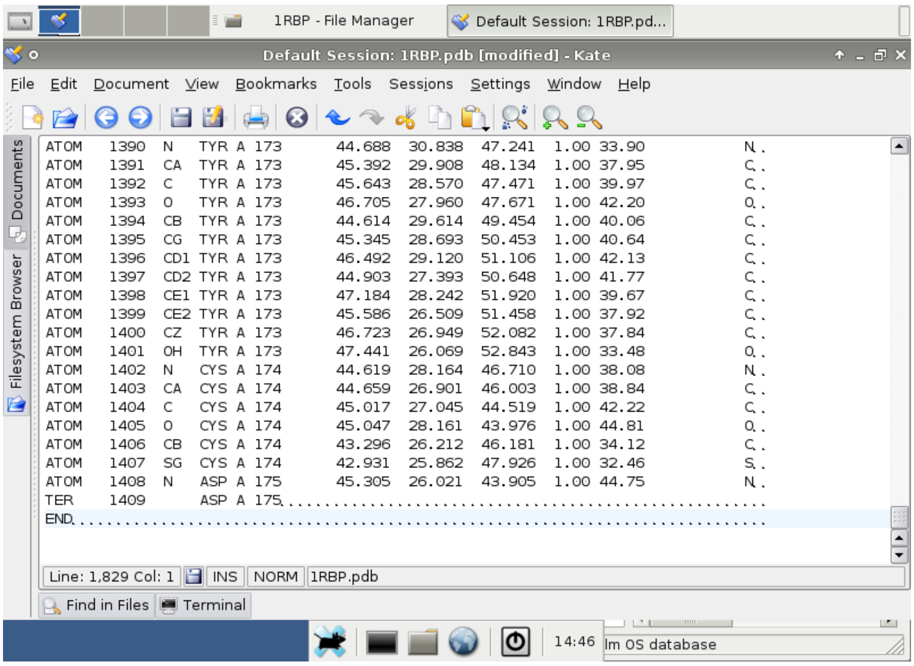

4. Save this file with a name like 1RBP_receptor.pdb on the 'home/user/tutorial-1RBP/protein/' folder (yellow arrow). Close the file.

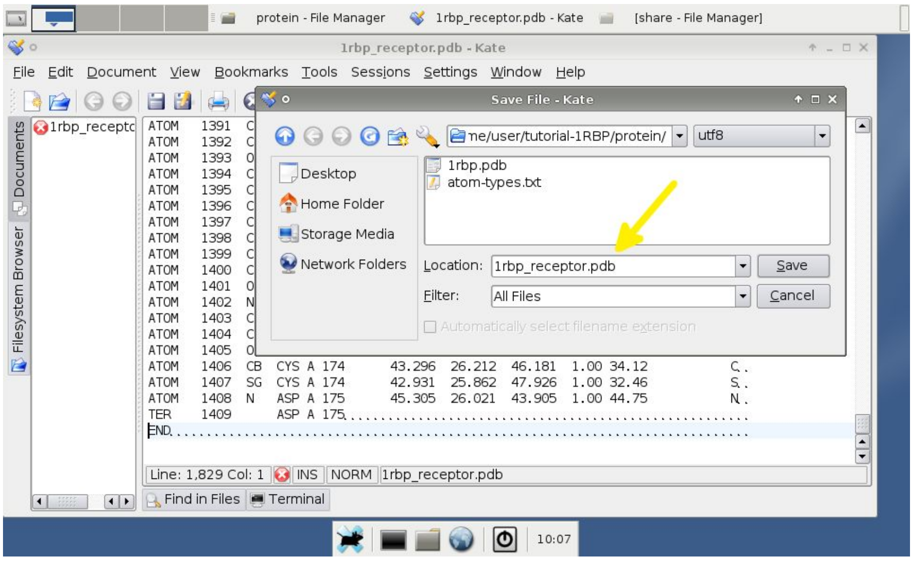

5. Next Re-open 1RBP.pdb and remove everything <u>except</u> the ligand Retinol (the quickest way to do this is to: copy Retinol, then delete all the text and then paste Retinol). Then save this file with a name like 1RBP_ligand.pdb. Retinol atoms are indicated by the RTL abbreviation. This step is not needed if you don't want the ligand to be docked in the VS project but it is usually advised as the ligand is a good control for assessing the quality of AutoDock4 and Vina docking results.

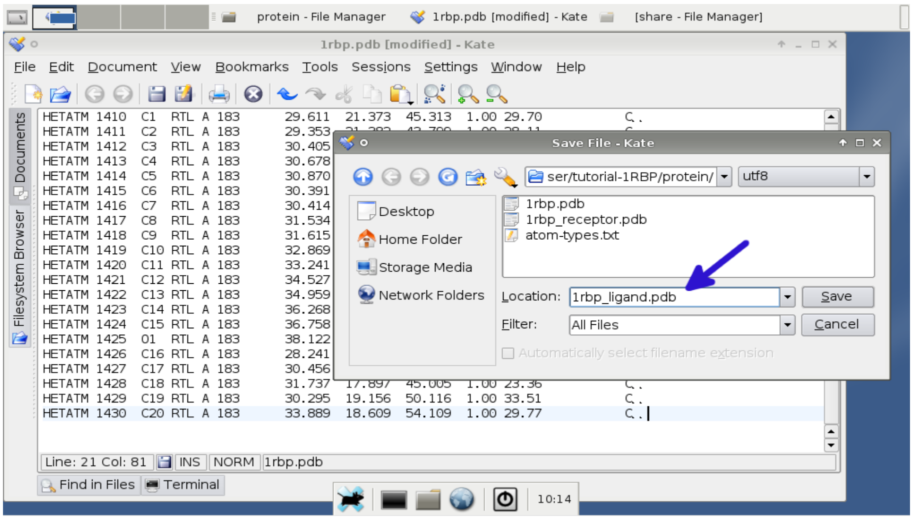

6. Close all text files, then open a Console (click the black box on the bottom) and type the command line 'runAdt' to open AutoDockTools. ADT is being opened from the CD so it may take a few moments. Select 'AutoDock 4.0' and click 'Dismiss'.

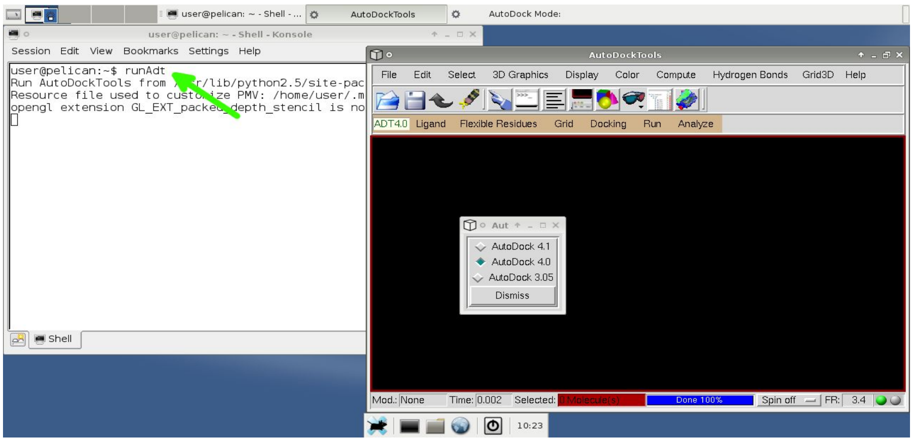

7. Now let’s prepare the protein receptor. The quickest way is to click on the 'Dashboard' button (orange arrow). Then click on the right mouse button on ‘PMV Molecules’ (pink arrow) and open the 1RBP_receptor.pdb file recorded on step 4 (brown arrow).

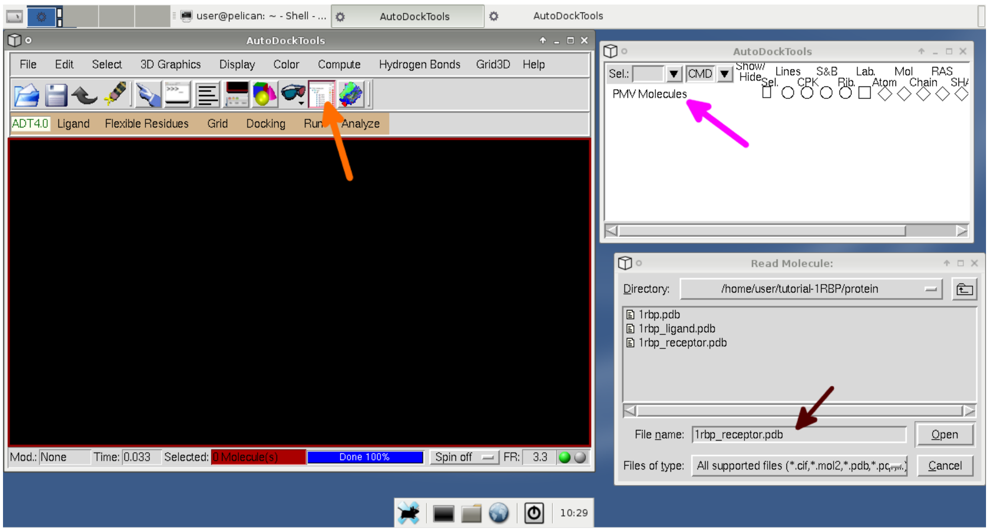

8. Then click on ‘Edit>Hydrogen>Add’ menu. Select the ‘All Hydrogen’, ‘noBondOrder (for PDB files…)’ and the ‘Yes’ options. PDB files don't usually have hydrogens but we need them for AutoDock4 and VINA so this step adds them.

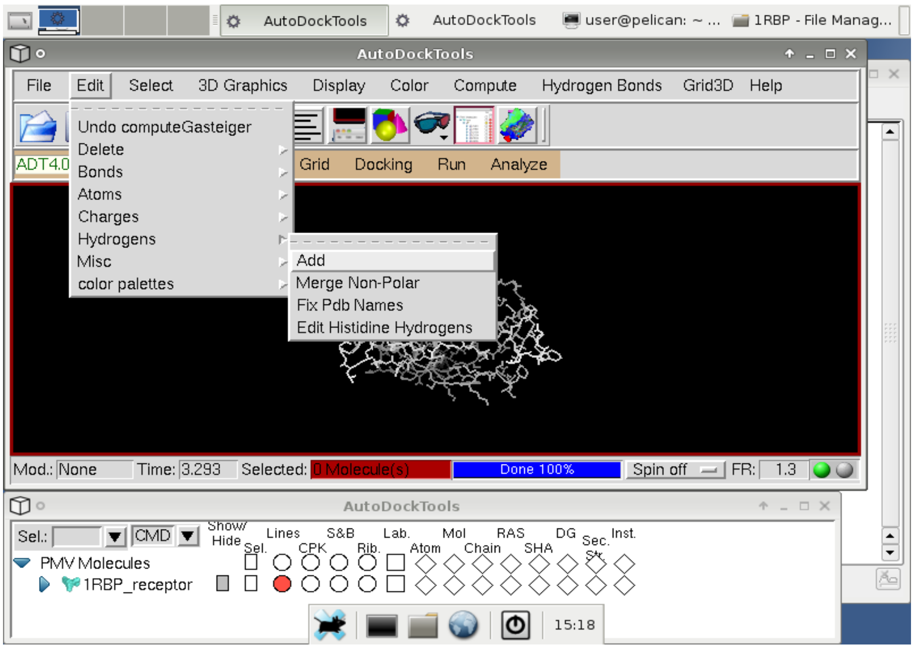

9. Then click on the ‘Edit>Charges>Compute Gasteiger’ menu to add charges to all atoms. If you don't have experience with ADT play around with the protein structure to familiarize yourself with the software.

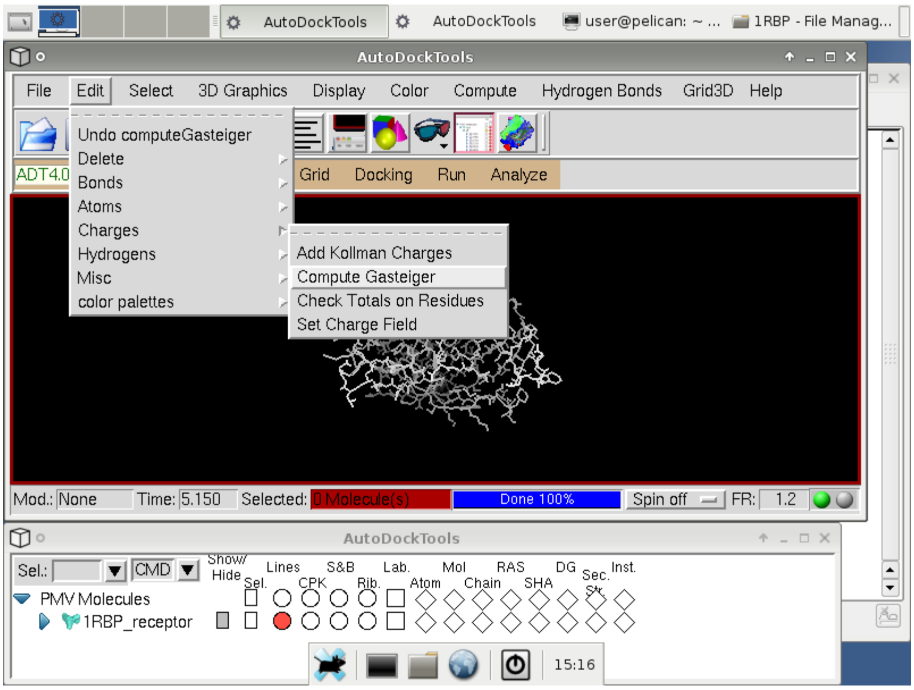

10. Finally click on the ‘Edit>Atom>Assign AD4 type’ menu.

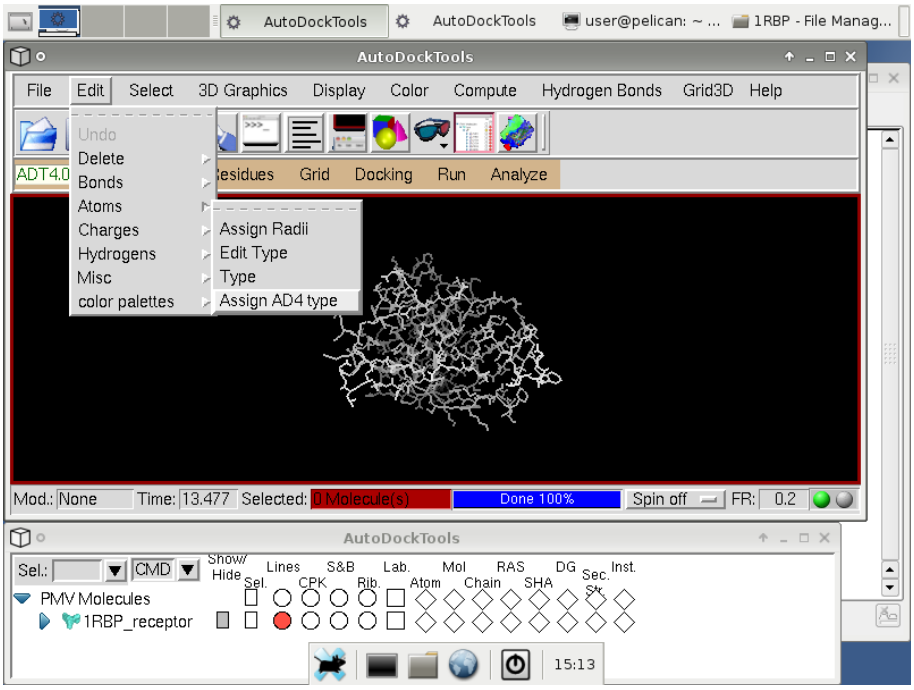

11. Now save the protein receptor in the PDBQT file format need for AutoDock4 and Vina. Click on the 'File>Save>Write PDBQT' menu.

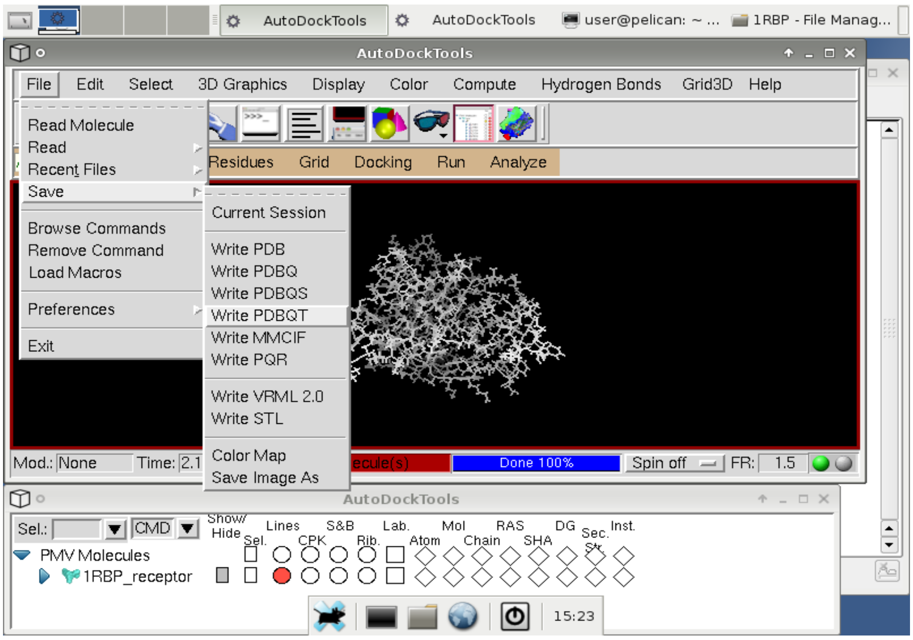

12. Don't forget to save it in the PROTEIN folder (red arrow), in this tutorial it's the 'home/user/tutorial-1RBP/protein/' folder. Click on the 'Browse' button, go to the project folder and use a file name like 1rbp_receptor.pdbqt.

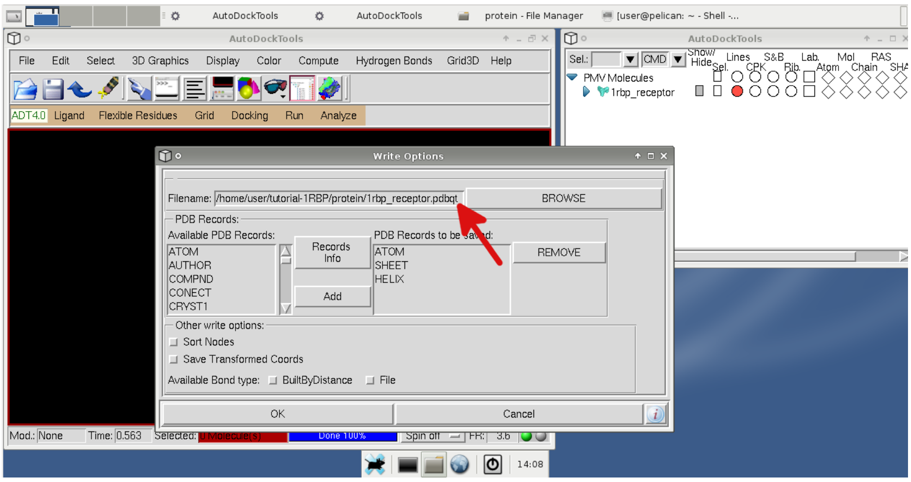

13. Now we are going to prepare the ligand (retinol). Click on the ‘Ligand>Input>Open’ menu (blue arrow). Then choose the 1rbp_ligand.pdb file recorded before from the PROTEIN folder (yellow arrow). You need to select the ‘pdb files (*.pdb)’ option on the ‘Files of type’ menu.

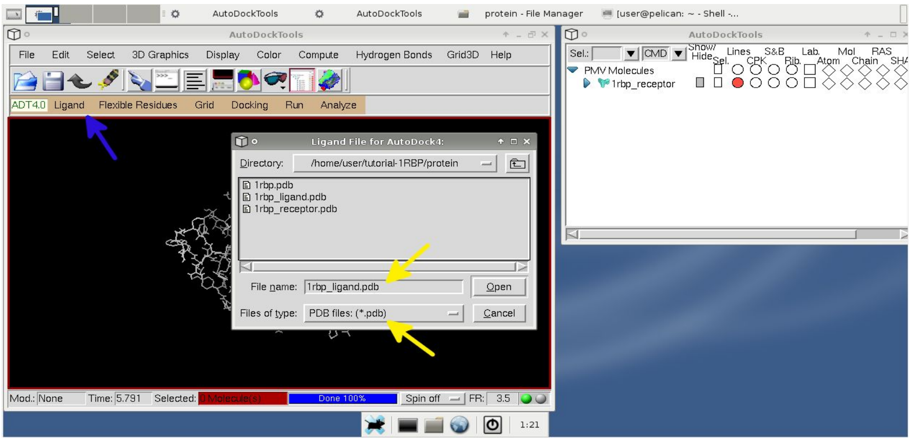

14. The retinol molecule opens on the display window. To save the ligand on the required PDBQT file format click on ‘Ligand>Output>Save as PDBQT…’ menu (orange arrow) and save the ligand on the tutorial-ligands folder 'home/user/tutorial-1RBP/ligands/tutorial-ligands' folder (pink arrows) using a name like ‘1rbp_ligand.pdbqt’. On the tutorial-ligands we already have 49 compounds of the NCI Diversity set II compound dataset to test MOLA. It's also available the complete NCI_Diversity set II in PDBQT format, totalling 1542 compounds. To see only the ligand you can use the dash board were you have several option to manipulate the molecules on the display (brown arrow). Quit ADT.

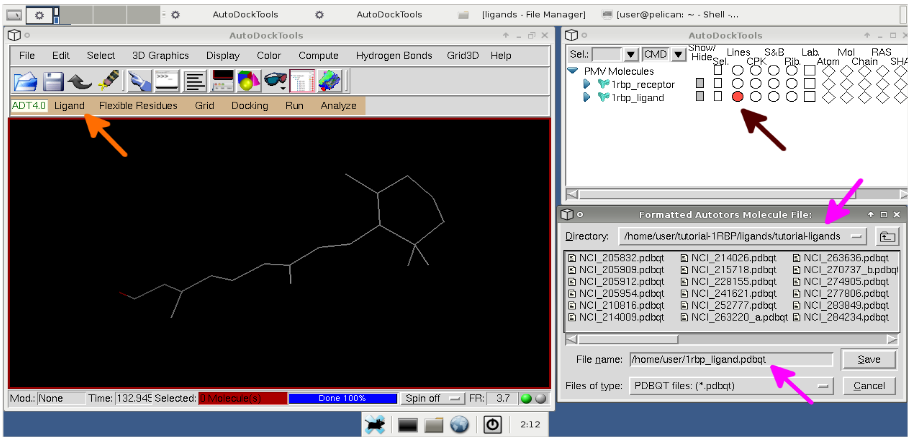

15. Now we have the protein and ligand files on the appropriate PDBQT format. For AutoDock4 we need to make atom grid maps that can be created with AutoGrid4 software. On our system AutoGrid4 only works on the '\home\user' folder so we need to transfer a copy a 1rbp_receptor.pdbqt file created before on the PROTEIN folder (red arrow) to the '\home\user' folder (blue arrow). Just drag-and-drop the file.

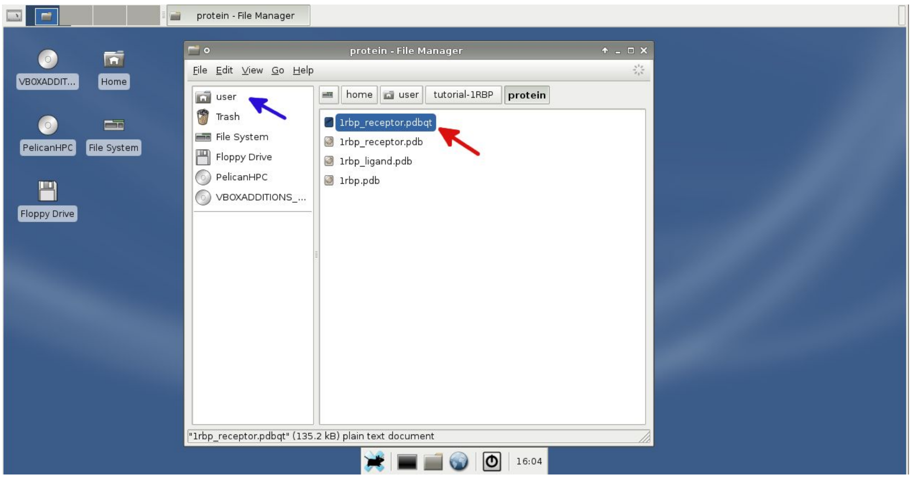

16. To create the atom grid maps re-open ADT, click on the 'Grid>Macromolecule>Open…' (yellow arrow) menu and select 1rbp_receptor (green arrows). The protein opens. Click 'Yes' to the windows that appear.

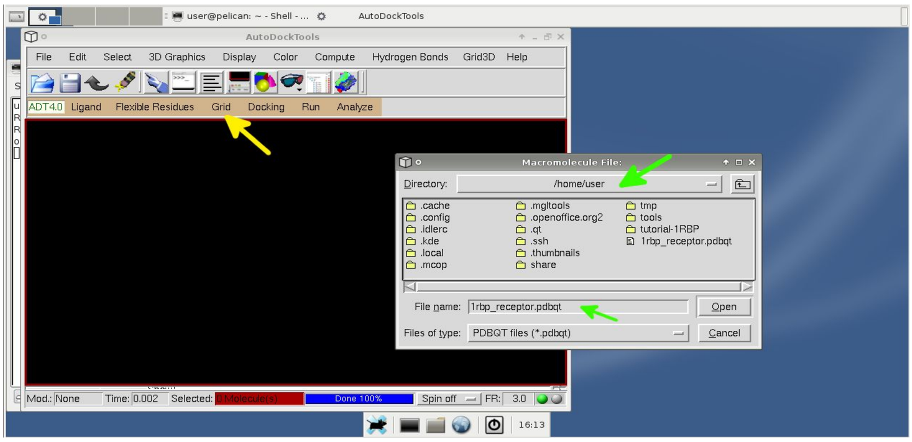

17. Then click on ‘Grid>Set Map Types>Directly…’ (orange arrow). Now we select the atom grid maps we want ADT to generate. Because we are using many compounds we generate atom grid maps for all the possible atom types. The atom types are the following 29:

A BR Br C CA Ca CL Cl F FE Fe H HD HS I

MG Mg MN Mn N NA NS OA OS P S SA ZN Zn

As ADT can only make 20 maps at a time we will make the atom maps in two AutoGrid4 steps. Copy paste the atom types from the first line (A to I) (pink arrow). A file called atom_types.txt is available on PROTEIN folder if you want to copy paste the atom types directly (brown arrow).

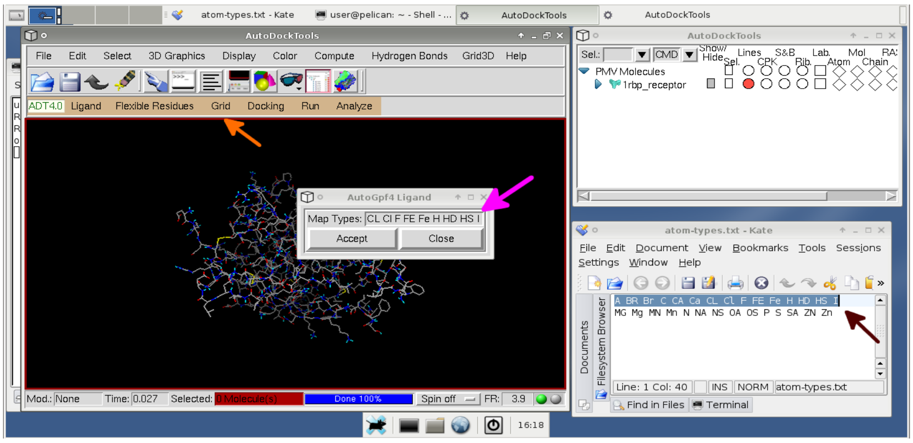

18. Now select ‘Grid>Grid Box’ menu to open the 'Grid options' window (red arrows). To select the 'Center Grid Box' on retinol open the 1rbp_ligand.pdbqt recorded before (blue arrow), then click on the 'Center>Pick an atom' menu (yellow arrow) and click an atom of retinol on the display window. The center of the grid will re-center on the selected atom. Size and position of the Grid depends on the protein structure used and the objective of each study. Then click on the ‘File>Close saving current’ menu to save the grid settings (green arrow).

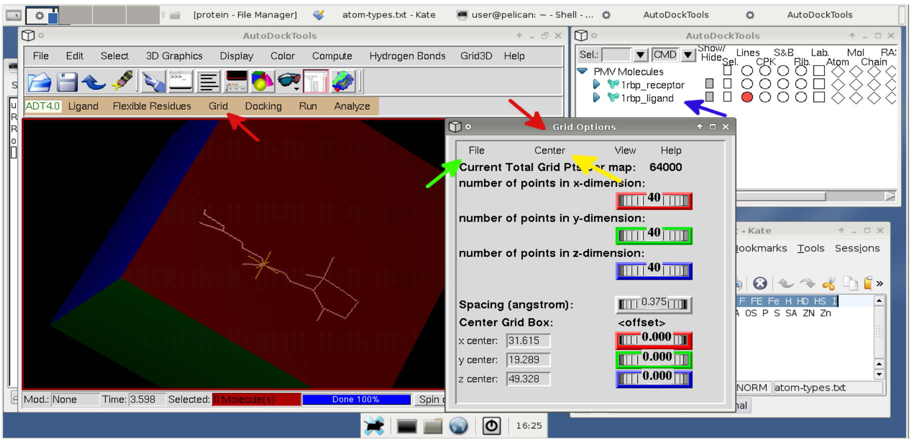

19. Then click on the ‘Grid>Output>Save GPF…’ menu and save a gpf (grip parameter file) file on the PROTEIN folder with a name like 1RBP.gpf (red arrow).

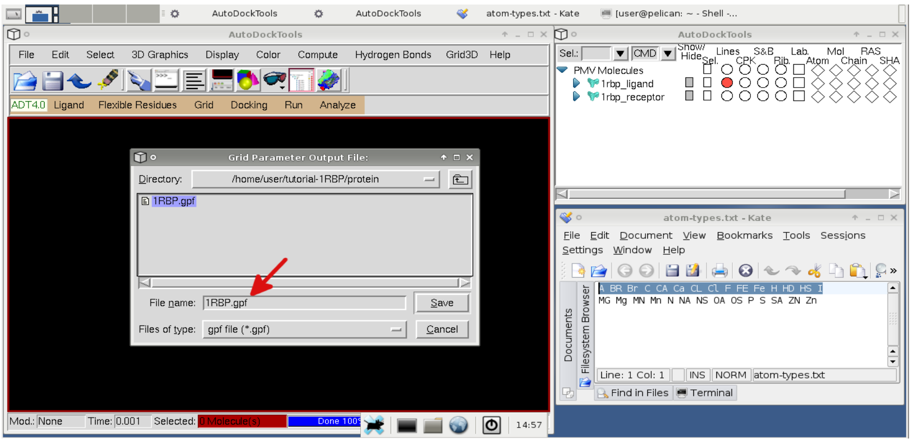

20. Finally we start AutoGrid4 to generate the atom grid maps. Click on the ‘Run>Run AutoGrid…’ menu (pink arrow). If everything is OK click ‘Launch’ (brown arrow).

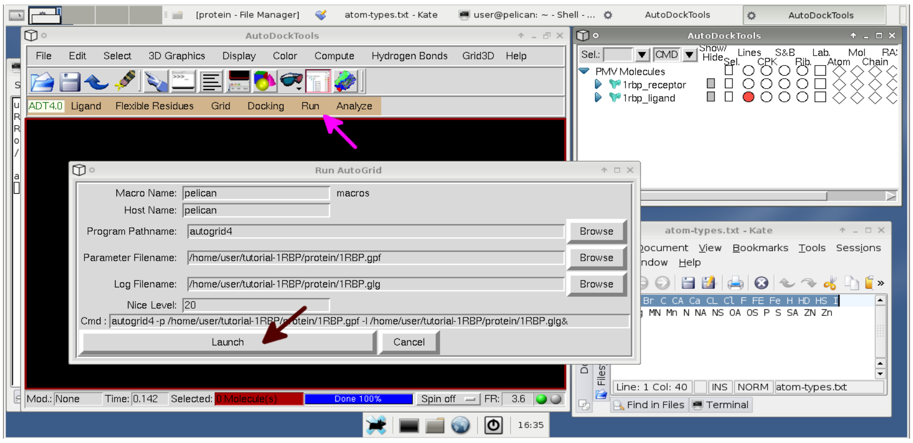

21. An ‘AutoDock Process Manager’ window appears and you can monitor atom grid map creation.

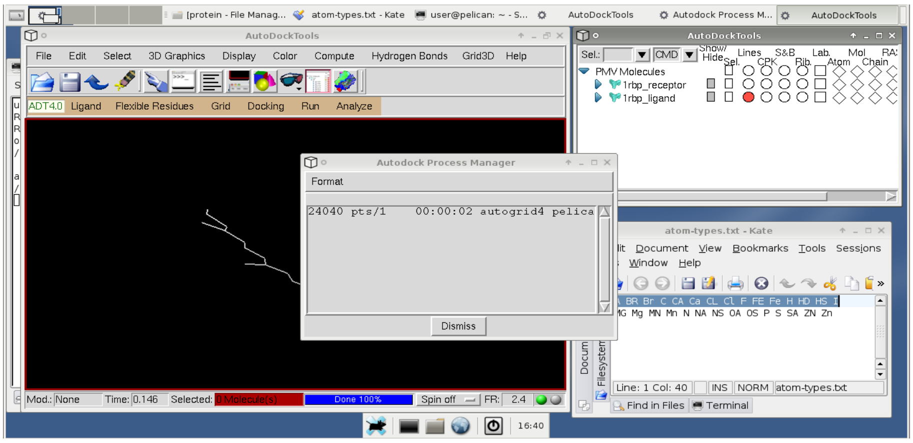

22. Check on the Shell Console for the ‘autoGrid4: Successful Completion’ message indicating that the atom grid maps are complete. By now you should see the atom grid maps on ‘/home/user’ folder. Repeat steps 17 through 21 but for the second row of atom types in order to have all the 29 atom type maps. The maps are created on the '/home/user' folder, so you need to transfer them to the PROTEIN folder (red arrow). This step is not mandatory but remember that the atom grid map files are on transient RAM memory and if your computer turns of for any reason the files disappear. It’s always a good idea to use a USB-flash drive. Also if all files needed for the next step of Virtual Screening are placed on the same project folder the organization will be simpler. Now you have all files needed for using MOLA with AutoDock4 and Vina.

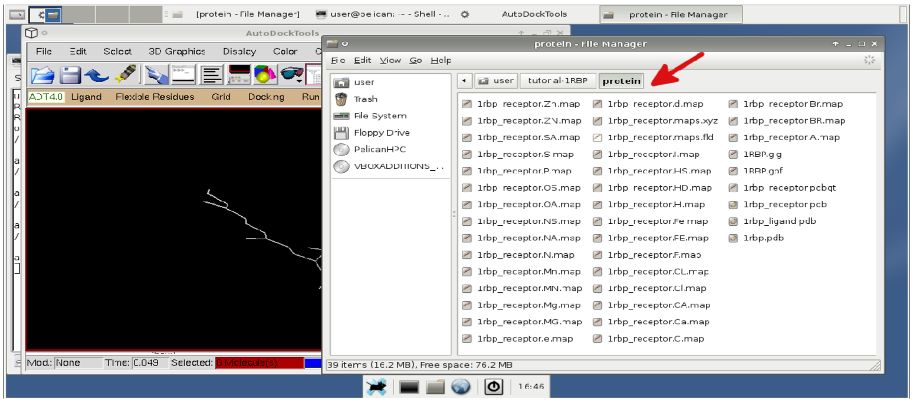

APPENDIX 2. Using VirtualBox for booting

The standard way yo use MOLA is by CD booting using the MOLA Live-CD burned from the available MOLA.iso file. However you can also use VirtualBox to boot MOLA without burning a CD and without restarting your master computer. In this case you just need to download the MOLA.iso file and follow this Appendix instructions. This options seems to work well for me although it was not extensively tested. Advantages for using VirtualBox are: (1) saving a CD, (2) possibility to capture sessions so that you can immediately restart were you left off, (3) you are able to continue to use your computer while doing a VS project and finally (4) you have the possibility to use the hard-disk instead off a USB-flash drive. The main disadvantage is diminished performance.

1. Go to the VirtualBox site http://www.virtualbox.org/ and download the appropriate version. There are versions for Windows, Macintosh and Linux. For example this tutorial was done on a Ubuntu (Linux) system with VirtualBox installed and MOLA system started from the MOLA.iso file. Once you have installed VirtualBox open it, you should have the following screen whatever operating system you use. Some parts are in Portuguese, sorry about that!

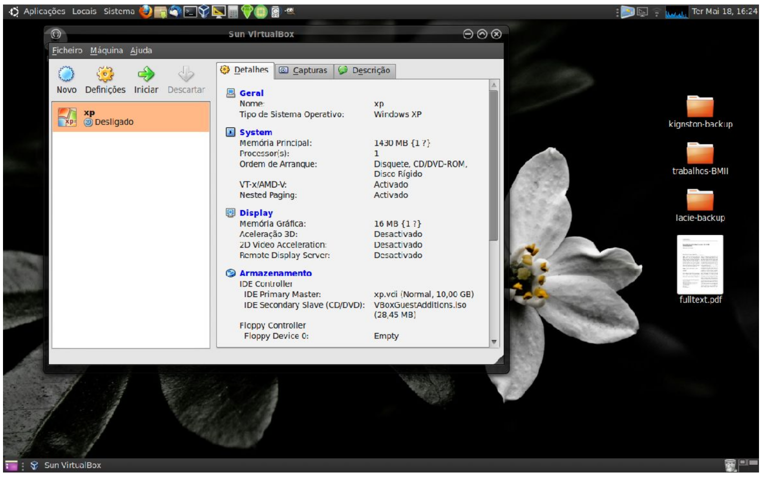

2. Click on 'New' icon and create a new virtual machine. I named it MOLA and selected as operating system Linux-Debian as MOLA is based on the Debian Live Distribution.

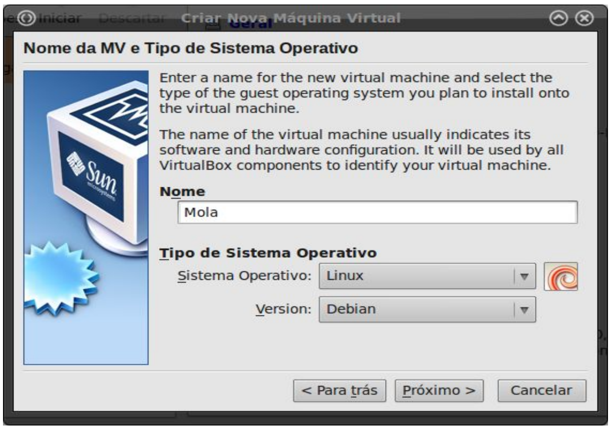

3. Then select the RAM memory you want use. 512 MB is the minimum value when using MOLA but 1024 MB is advisable. For your master computer node you should use the more powerful computer with more RAM memory. You can assign to VirtualBox up to half the RAM memory with confidence. Click 'Next'.

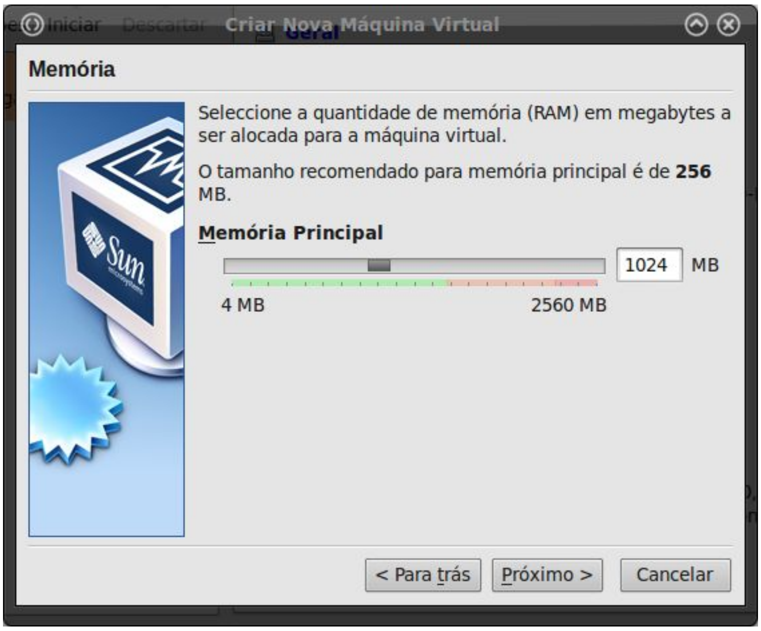

4. Select 'boot hard-disk (Primary Master) > Create new hard disk, then select the 'dynamically expanding storage' option and the 8 Gb default value for the MOLA hard-disk. You should see the next screen. Click 'Finish'.

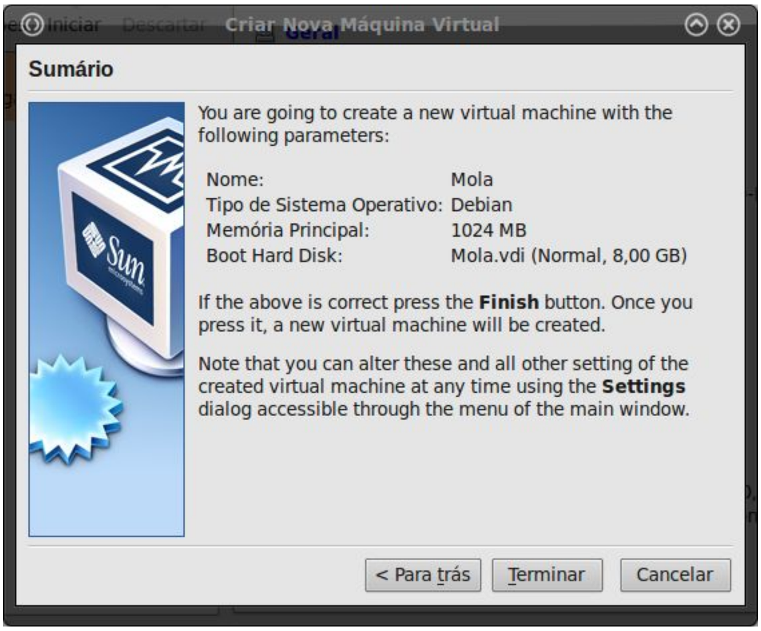

5. The Mola system now appears on VirtualBox. Click the 'Start' icon, Mola system starts on a new window. Now a screen asks for the boot device. Click the icon shown by the blue arrow.

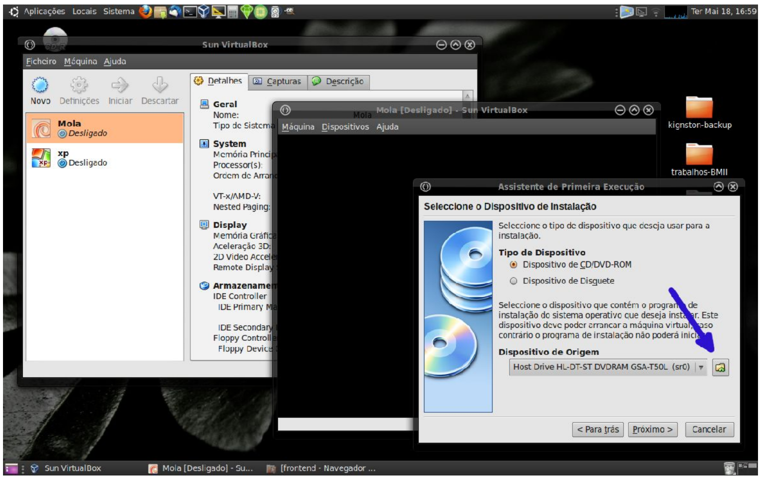

6. Click the 'Add' (yellow arrow). You will be asked to selected a ISO file, select the MOLA.iso file you downloaded earlier. The MOLA.iso file should now appear on the window (green arrow). Make sure to add the VBoxGuestAdditions.iso as this will be important in a few moments. Now click on the' Select' button.

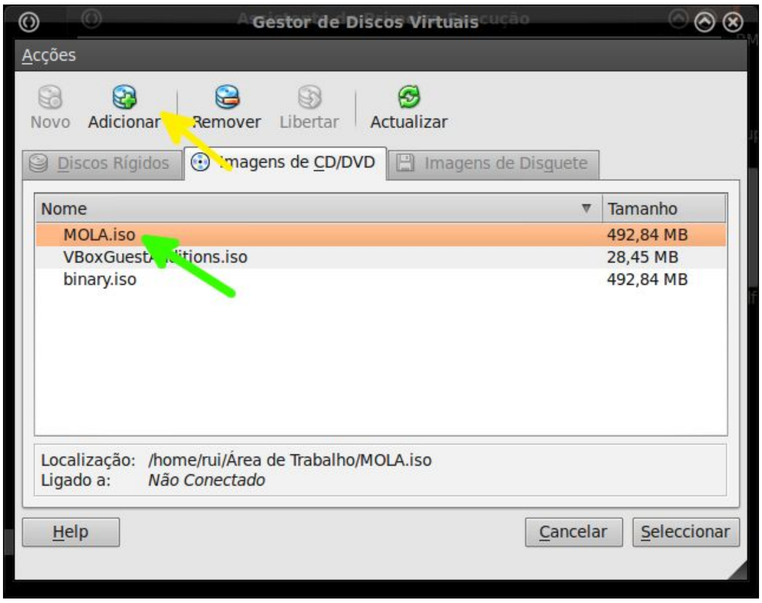

7. Now MOLA system starts just has it would start if you booted from the CD like it's described on PART1 of this tutorial.

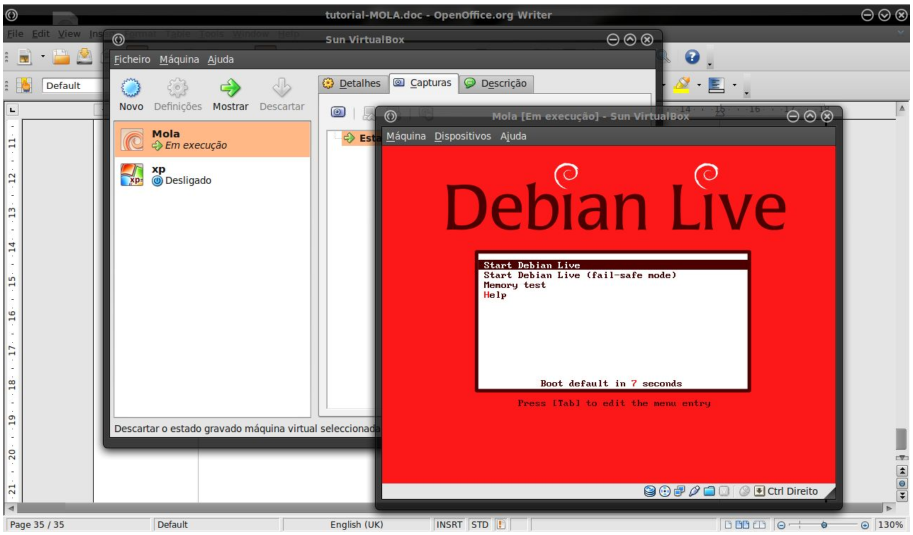

8. Follow the same instructions until the MOLA system has started, you should see a windows like this. From this point you can assemble the cluster as described on PART 1 of this tutorial. However a few more steps are described that facilitate the use of MOLA with Virtual-box.

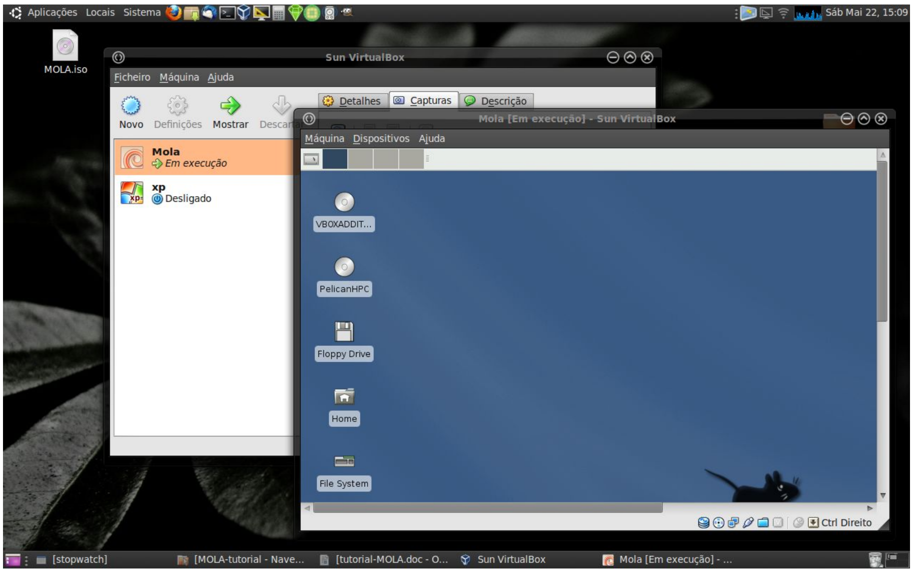

9. We will now install the VirtualBox Guest Additions tools. This tools are important for 3 reasons: (1) allows auto-scaling of MOLA window, (2) MOLA window will work seamlessly with the host system (no need to press Ctrl-Alt-Del to leave from MOLA window to the host system) and more importantly (3) allows the creation of a link to access the host file system (host hard-disk drive). First double click on the VBOXADDITIONS_3.1.6.59338 icon on the Desktop. Then open a Terminal (click the mouse right button and select 'Terminal' option) and type this command (red arrow):

'sudo sh /media/VBOXADDITONS_3.1.6.59338/VBoxLinuxAdditions-x86.run'

After a moment the VirtualBox Additions are installed.

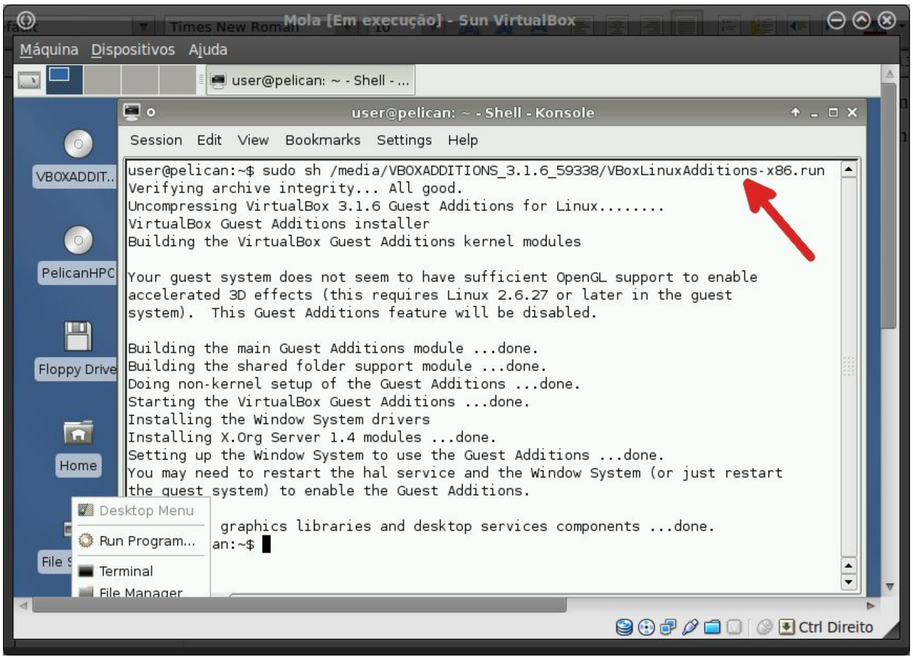

10. To enable VirtualBox Additions you have to 'log out' the system (blue arrows) then restart writing 'startx' at the command line.

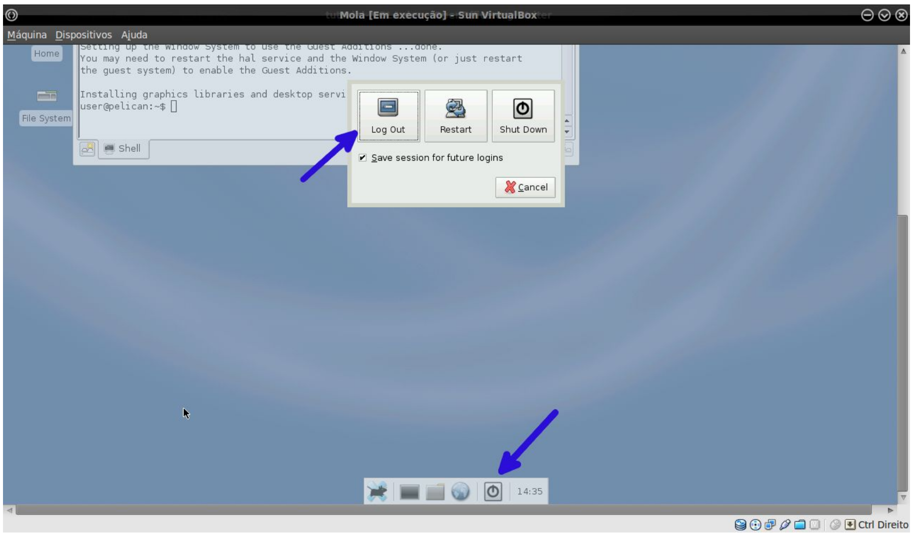

11. Now we are going to create a link to the host system. Create a 'share' folder on 'home/user/' folder (we name it share but you can use any other name (yellow arrow).

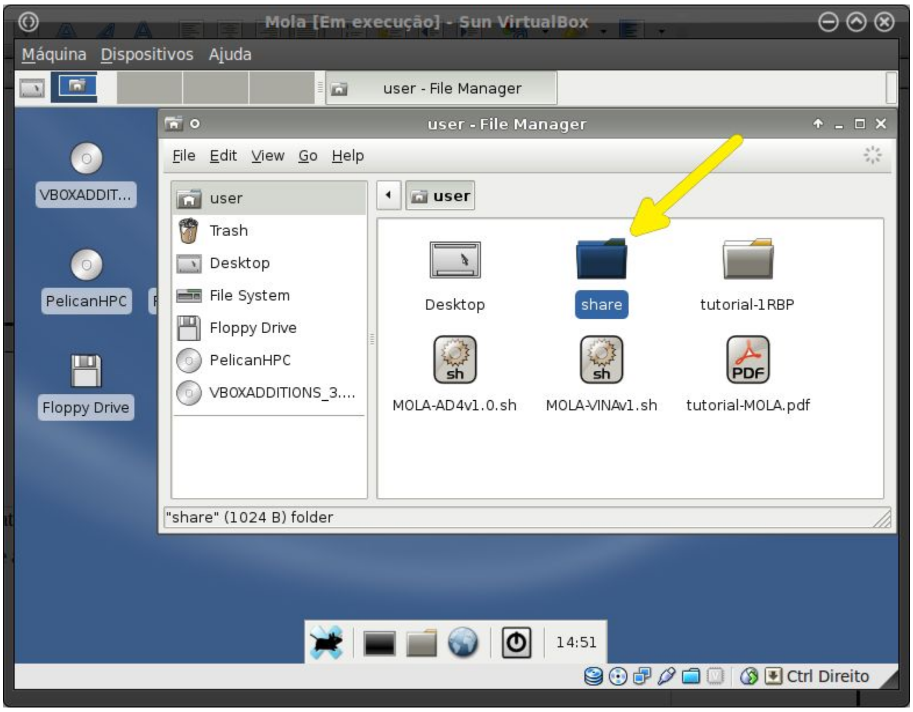

12. Open the 'Device>Share Folders...' menu.

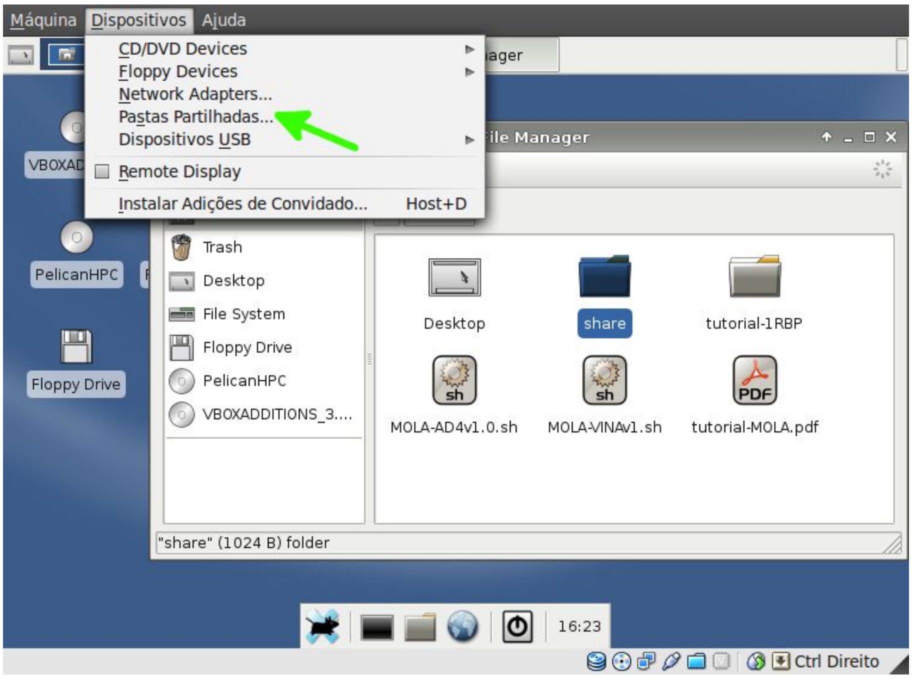

13. On the 'Shared folders' window click the 'Add' button (black arrow) and select the folder (red arrow) from the host you would like to share (in this example the '/home/rui' folder) and give a name to it (in this example the name is 'share'). Click 'OK'

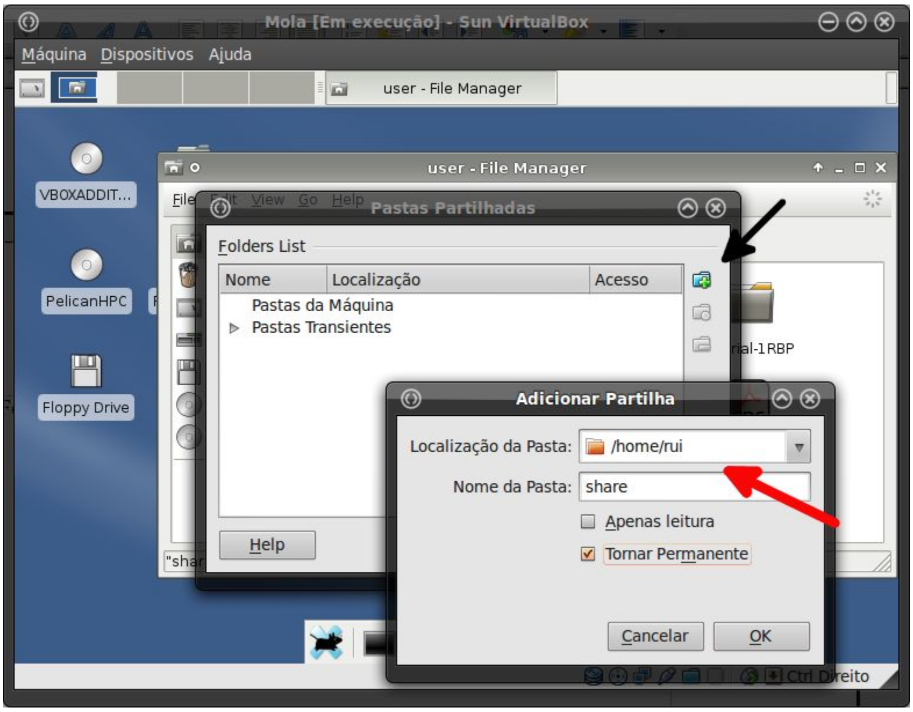

14. Open a Terminal and type this command (blue arrow):

sudo mount -t vboxsf -0 uid=1000 share /home/user/share

Now if you open the '/home/user/share' folder you have a direct link to the '/home/rui' folder from the host system (orange arrow). You can use this folder as any other folder from the system. What this means is that you can select the input files for your VS project from on a PROJECT folder on the the host hard-disk drive instead of on a USB-flash drive. And of course the results files will also be recorded there.

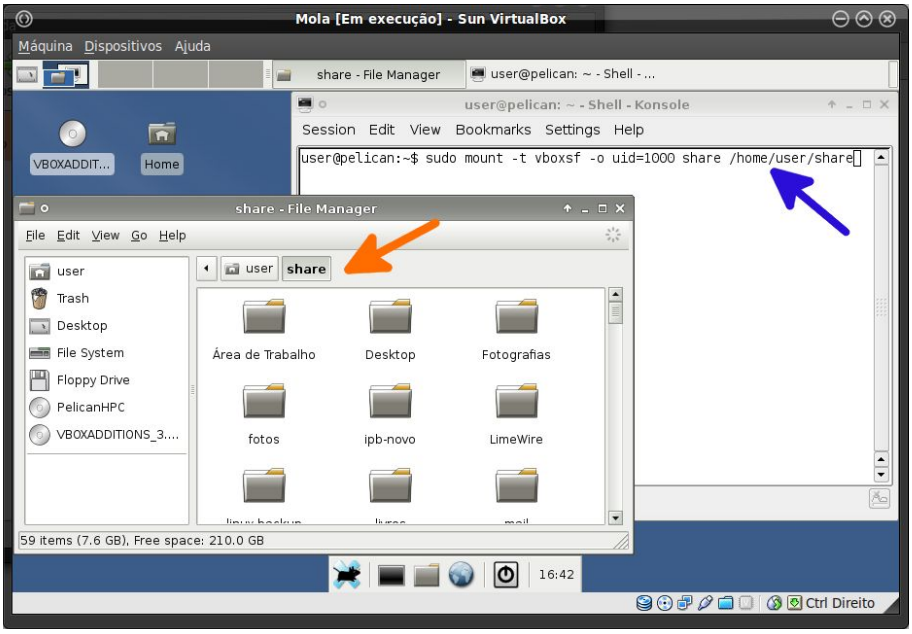

15. Only one step is missing. Open the 'Devices>Network Adaptor...' menu (green arrow). Check if 'Adaptor 1' is linked to your host ethernet device (usually 'eth0') and that the 'Bridged adaptor' option is selected (yellow arrow). Click 'OK'. This creates a virtual link to your ethernet connector that will control the cluster.

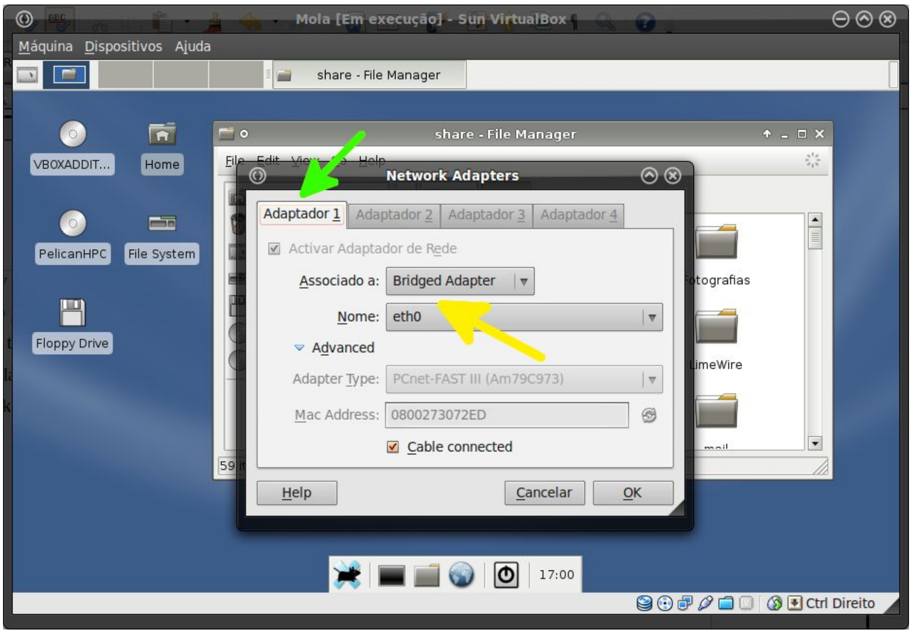

16. At this point you are ready to start PART 2 and 3 of this tutorial and use MOLA. The difference is that you can launch your VS project with MOLA inside a Virtual-Box while using your computer for other tasks. Also with the link(s) we set up to the host we can select the input files and record the output result files directly to the host hard-disk drive. The downside of this system is diminished performance as the computer resources are divides by the host and the guest (MOLA) system.

Important! If you're host system has DHCP connection enabled, please disconnect it when using MOLA, or at least it shouldn't be connecting automatically (red arrow). The reason is that when assembling the cluster (PART 1) the host will be detected as one slave node and the system will not work.
